# Quantification of nematic cell polarity in three-dimensional tissues

**DOI:** 10.1371/journal.pcbi.1008412

**Published:** 2020-12-10

**Authors:** André Scholich, Simon Syga, Hernán Morales-Navarrete, Fabián Segovia-Miranda, Hidenori Nonaka, Kirstin Meyer, Walter de Back, Lutz Brusch, Yannis Kalaidzidis, Marino Zerial, Frank Jülicher, Benjamin M. Friedrich

**Affiliations:** 1 Max Planck Institute for the Physics of Complex Systems, Dresden, Germany; 2 Centre for Information Services and High Performance Computing, TU Dresden, Dresden, Germany; 3 Max Planck Institute of Molecular Cell Biology and Genetics, Dresden, Germany; 4 Institute for Medical Informatics and Biometry, Faculty of Medicine Carl Gustav Carus, TU Dresden, Dresden, Germany; 5 Center for Advancing Electronics Dresden, TU Dresden, Germany; 6 Cluster of Excellence Physics of Life, TU Dresden, Germany; 7 Institute for Theoretical Physics, TU Dresden, Germany; University of Michigan, UNITED STATES

## Abstract

How epithelial cells coordinate their polarity to form functional tissues is an open question in cell biology. Here, we characterize a unique type of polarity found in liver tissue, nematic cell polarity, which is different from vectorial cell polarity in simple, sheet-like epithelia. We propose a conceptual and algorithmic framework to characterize complex patterns of polarity proteins on the surface of a cell in terms of a multipole expansion. To rigorously quantify previously observed tissue-level patterns of nematic cell polarity (Morales-Navarrete et al., eLife 2019), we introduce the concept of co-orientational order parameters, which generalize the known biaxial order parameters of the theory of liquid crystals. Applying these concepts to three-dimensional reconstructions of single cells from high-resolution imaging data of mouse liver tissue, we show that the axes of nematic cell polarity of hepatocytes exhibit local coordination and are aligned with the biaxially anisotropic sinusoidal network for blood transport. Our study characterizes liver tissue as a biological example of a biaxial liquid crystal. The general methodology developed here could be applied to other tissues and in-vitro organoids.

## Author summary

Cell polarity enables cells to carry out specific functions. Cell polarity is characterized by the formation of different plasma membrane domains, each with specific composition of proteins, phospholipids and cytoskeletal components. In simple epithelial sheets, or tube-like tissues such as kidney, epithelial cells are known to display a single apical domain, facing a lumenal cavity, and a single basal domain on the opposite side of the cell, facing a basal layer of extracellular matrix. This apico-basal polarity defines a vector of cell polarity, which provides a direction of fluid transport, e.g., from the basal side of the sheet to the lumen-facing side. In more complex, three-dimensional epithelial tissues, such as liver tissue with its complex network of blood-transporting sinusoids, the membrane domains of hepatocyte cells display more intricate patterns, including rings and antipodal pairs of apical membrane. Here, we develop a mathematical framework to precisely characterize and quantify complex polarity patterns. Thereby, we reveal ordered patterns of cell polarity that span across a liver lobule. Our new method builds on physical concepts originally developed for ordered phases of liquid crystals. It provides a versatile tool to characterize the spatial organization of a complex three-dimensional tissue.

## Introduction

In multi-cellular organisms, almost all tissue cells are spatially asymmetric to serve their function inside their host tissue [1]. This *cell polarity* can be realized by different kinds of physical anisotropies, including cell shape, the structural polarity of their cytoskeleton [2], or the protein and lipid composition within the cell membrane [3, 4].

Here, we focus on the anisotropic distribution of functional membrane domains on the surface of cells, and use the term *cell polarity* specifically for this important case. A prototypical example is the distribution of polarity-specific apical and basal membrane proteins on the surface of epithelial cells [1].

**Fig 1:**
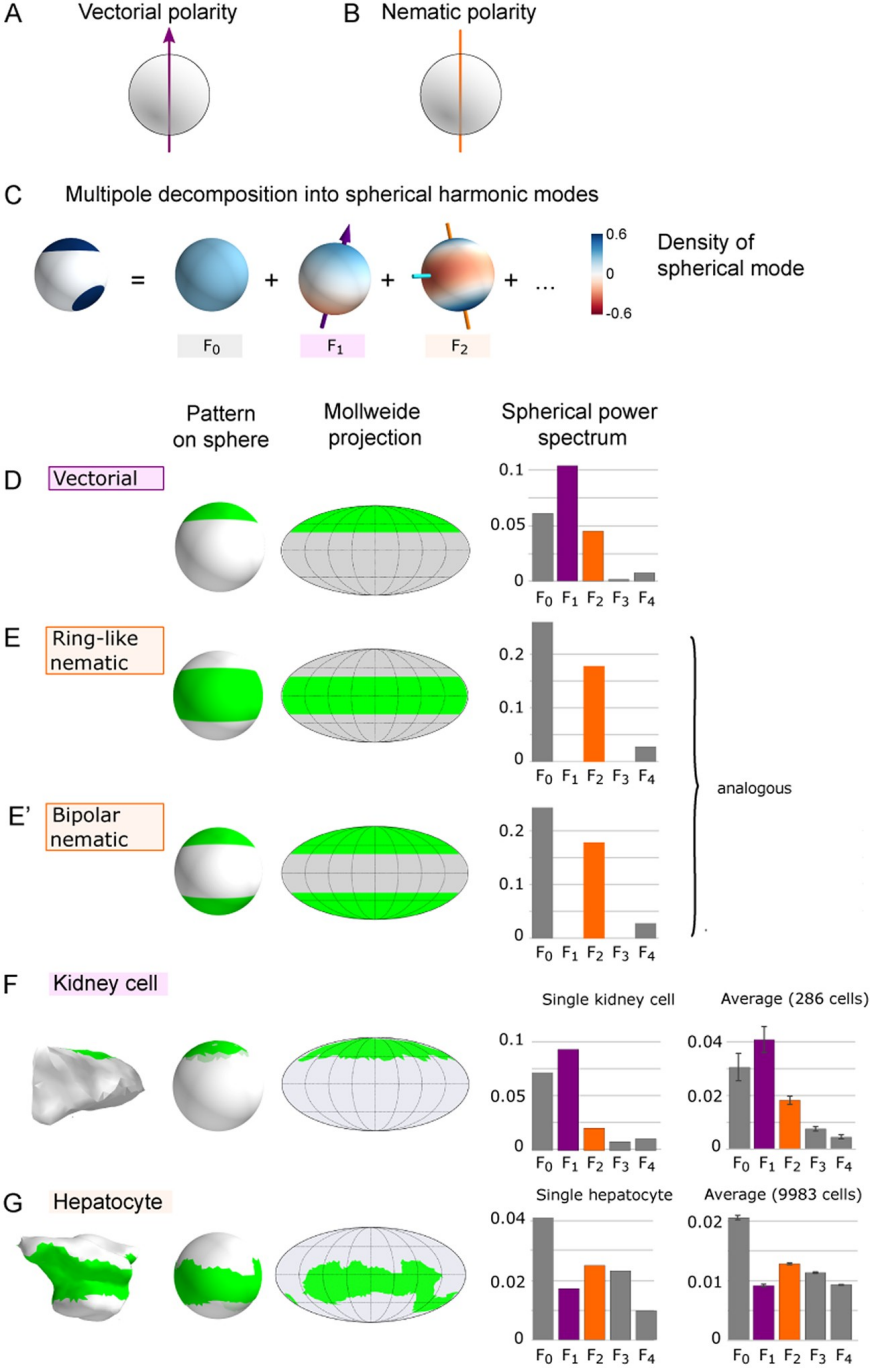
Multipole decomposition of surface patterns. (A, B) Schematic of vectorial and nematic cell polarity, respectively. (C) Multipole decomposition of a distribution on a sphere into spherical harmonics, see Eq (1). (D) Prototypical membrane distribution of vectorial polarity type with respective Mollweide projection and spherical power spectrum. The spherical power spectrum shows the power ||*F*_*i*_||^2^ of the each mode *F*_*i*_ corresponding to the contribution from spherical harmonics of order *i*, *i* = 0, 1, 2, 3, 4, see Eq (2). (E) Same as panel D but for a ring-like surface distribution. Here, the second mode of the spherical power spectrum dominates. (E’) The second mode of the spherical power spectrum also dominates for the analogous case of a bipolar surface distribution. (F) Spherical projection, Mollweide projection and spherical power spectrum for an epithelial tubular cell from kidney tissue, as well as averaged power spectrum for a population of cells (*n* = 286, mean±2 ⋅ s.e.m., corresponding to 95% confidence interval). (G) Same as panel F, but for a hepatocyte from mouse liver tissue, as well as a population of hepatocytes (*n* = 9983).

Among the main functions of epithelial tissues are absorption, filtration, and transport of macromolecules [1]. Simple epithelial tissues usually cover a body surface or line a body cavity and consist of a one-cell thick layer of cells. Specifically, apical domains form on the luminal side of the tissue that faces the cavity. Apical domains are separated by tight junctions from other membrane domains, such as lateral and basal domains. Lateral domains provide cell-cell adhesion, while basal domains form the interface with the basement membrane and extracellular matrix [3, 4]. This structural asymmetry of apical and basal domains in simple epithelia defines a vectorial cell polarity (sometimes referred to as *columnar polarity* [4]), see Fig 1A. This vectorial cell polarity sets a direction for the *directed transport* of macromolecules.

However, there are also epithelial tissues with a more complex, three-dimensional architecture, such as liver tissue [3–7]. The functional unit of the liver, the liver lobule, is organized around a central and a portal vein, which are connected by a dense, three-dimensional network of sinusoids that transport blood (see also Fig 4A). Hepatocytes, the main cell type of the liver, are evenly distributed in the lobule with a volume fraction of approximately 80% [7]. Each hepatocyte is in contact with the sinusoidal network at multiple basal membrane domains, which facilitate the exchange of metabolites with the blood stream [4]. The sinusoidal network was proposed to provide orientational cues to hepatocytes [8, 9]. In addition to the basal contacts, each hepatocyte possesses multiple apical membrane domains that form narrow lumina with adjacent cells, into which bile is excreted [4, 10, 11]. These lumina form a second, three-dimensional network, the bile canaliculi network. The direction of bile excretion by individual hepatocytes and, correspondingly, the distribution of apical membrane domains on their surface, cannot be characterized by a single vector, yet is also not random.

Previously, Elias put forward an idealized description of liver tissue in terms of a crystal-like organization of sinusoids and polarized hepatocytes [12, 13]. The Elias model has recently been revisited using high-resolution imaging data of mouse liver tissue [14]. In that study, it was shown that the structure of liver tissue is intermediate between an amorphous structure and a perfect crystal, best described as a liquid crystal with orientational but not positional order. The quantification of cell polarity in three-dimensional reconstructions of such high-resolution data prompts new analysis methods to infer the coordination of cell polarity at the tissue level.

We will characterize the distribution of apical membrane domains on the surface of hepatocytes by a tripod of nematic axes. Intuitively, a nematic axis can be thought of as a double-headed arrow that specifies an axis, but does not single out any of the two directions parallel to that axis, see Fig 1B. We will refer to this characterization as *nematic cell polarity* to highlight the analogy to *vectorial cell polarity* (although a set of nematic axes is not polar in the strict mathematical sense).

A first approach was restricted to the analysis of a single type of cell polarity axes at a time [15]. Here, we extend the analysis in [15] to the biaxial case of a full tripod of nematic cell polarity axes. We present a systematic and versatile method to characterize cell polarity by means of a multipole expansion. The zeroth moment of this expansion describes a uniform surface density of a polarity marker, as found, e.g., in non-polarized mesenchymal cells. The first moment of this expansion describes vectorial polarity, and characterizes, e.g., apico-basal polarity of cells in simple epithelial sheets. The second moment defines nematic cell polarity, and characterizes, e.g., the more complex distribution of apical membrane domains found in hepatocytes.

We apply the concept of nematic cell polarity to apical membrane patterns of hepatocytes. We find that the nematic cell polarity of hepatocytes is aligned along curved director fields within the liver lobule, in line with previous observations [15]. We demonstrate that the coordination of cell polarity is biaxial, i.e., its description requires two local reference axes. Additionally, we find that nematic cell polarity of hepatocytes is correlated with the local biaxial anisotropy of the sinusoidal network. A minimal interaction model conceptualizes the co-alignment of hepatocyte cell polarity and the local anisotropy of the sinusoidal network.

The co-orientational order parameters (COOP) introduced here to characterize the structure of three-dimensional liver tissue naturally generalize previous work on order in effectively two-dimensional cells and tissues. Drew et al. introduced COOP for two-dimensional systems, and applied this analytical metric to quantify the co-alignment of cytoskeletal structures in muscle cells [2]. Other authors addressed planar cell polarity [16, 17], or nematic alignment of cell shape elongation [18]. The COOP introduced here provide a unified framework to characterize such cellular anisotropies also in three space dimensions.

## Results

### Nematic cell polarity

We present a method to classify distributions of polarity membrane domains on the surface of cells by a multipole expansion in terms of their spherical power spectrum. Using this spherical power spectrum, we describe the dominant symmetry of such a distribution of membrane proteins in terms of either predominantly vectorial, nematic or higher-order type. We first illustrate the method using distributions on a sphere, and afterwards show how surface distributions on cells of non-spherical shape can be mapped to this case. For the convenience of the reader, a list of mathematical symbols can be found in S1A Appendix.

Let *f*(**x**) with $x\in S^{2}$ represent an area density on the surface of the unit sphere $S^{2}$, i.e., a *surface pattern*. Similar to the two-dimensional Fourier transform for functions defined on a plane, we decompose the density *f*(**x**) into a sum of *spherical harmonic modes* *F*_*l*_(**x**), see also Fig 1C

$$\begin{array}{c} \underset{\text{surface}\;\text{density}}{\underset{︸}{f(x)}}=\sum_{l=0}^{\infty }\underset{\text{spherical}\;\text{harmonic}\;\text{modes}}{\underset{︸}{F_{l}\left(x\right)}}\;. \end{array}$$ (1)

Here, the spherical harmonic mode *F*_*l*_(**x**) of degree *l* is a linear superposition of spherical harmonics of degree *l*, and can be written as $F_{l}\left(x\right)=\sum_{m=-l}^{l}f_{l}^{m}Y_{l}^{m}\left(x\right)$, where $Y_{l}^{m}\left(x\right)$ denotes the spherical harmonic of degree *l* and order *m* (normalized to unity). The expansion coefficients $f_{l}^{m}$ are unique and can be computed as a scalar product on the unit sphere between the original density *f*(**x**) and the spherical harmonics $Y_{l}^{m}$ as $f_{l}^{m}=\int_{S^{2}}{\text{d}}^{2}xf(x)Y_{l}^{m*}(x)$. Here, integration is performed over the unit sphere $S^{2}$ (with respect to the standard Euclidean measure), while the star denotes the complex conjugate. This formula for the expansion coefficients $f_{l}^{m}$ follows from the ortho-normality of the spherical harmonics, i.e., $\int_{S^{2}}d^{2}x\;Y_{l}^{m}Y_{l^{\prime }}Y^{m^{\prime }*}=\delta_{ll^{\prime }}\;\delta^{mm^{\prime }}$.

A visual representation of this spherical decomposition is given in Fig 1C: the *zeroth mode* *F*_0_ is isotropic and encodes the mean of the surface distribution *f*(**x**). The *first mode* *F*_1_(**x**) can be represented by a vector that points to the spherical average of the surface distribution [19]. The *second mode* *F*_2_(**x**) is related to nematic polarity and will be at the focus of this work. In fact, the nematic axes of cell polarity as well as the co-orientational order parameters to be introduced in this manuscript depend only on this second mode *F*_2_(**x**). The possible existence of higher modes is indicated. The original distribution can be restored by summing up all modes.

In analogy to Fourier analysis of one-dimensional signals, we define the *spherical power spectrum* of the surface pattern *f*(**x**) as the sequence ||*F*_*l*_(**x**)||^2^ for *l* = 0, 1, 2, …, where ||*F*_*l*_(**x**)||^2^ denotes the squared *L*^2^-norm of each spherical mode *F*_*l*_(**x**) (normalized by the surface area of the unit sphere)

$$\begin{array}{c} \left|\right|F_{l}{\left|\right|}^{2}=\frac{1}{4\;\pi }\int_{S^{2}}d^{2}x\;{\left|F_{l}\right|}^{2}\;,\;l=0,1,2,\ldots \;. \end{array}$$ (2)

The spherical power spectrum defined in Eq (2) satisfies a generalized Parseval’s theorem $\|f{\|}^{2}=\sum_{l}{\left\|F_{l}\right\|}^{2}$, where ||*f*||^2^ = (4*π*)^−1^∫*d*^2^ **x**|*f*(**x**)|^2^, i.e., the total power of the density *f*(**x**) equals the summed power of its modes. The spherical power spectrum ||*F*_*i*_||^2^ is a true invariant of the pattern *f*(**x**), i.e., it does not depend on the choice of coordinate system (while the coefficients $f_{l}^{m}$ depend on this choice). Some authors denote the spherical power spectrum also by *S*_*ff*_(*l*) = ||*F*_*l*_||^2^.

Fig 1D and 1E show prototypical vectorial and nematic distributions and their respective spherical power spectra. We visualize surface patterns also as Mollweide projections, an equal-area, pseudocylindrical geographic projection [20]. The spherical power spectrum of the cap-like distribution, shown in Fig 1D, has a clear peak at the first mode, corresponding to a predominantly vectorial polarity type of the surface distribution. In contrast, for a ring-like pattern as shown in Fig 1E (and, analogously, for a bipolar pattern with two antipodal caps, see Fig 1E’), all odd modes of the spherical power spectrum, including the first mode, vanish by symmetry. The power spectrum attains its maximum at the second mode, which classifies these distributions as nematic.

Biological cells are not perfectly spherical. We propose a simple method to project distributions on the surface of star-convex shapes onto a sphere (i.e., we require that shape can be represented as distance from a common center, which is taken to be the origin, as a single-valued function of the solid angle). Our method allows analyzing the anisotropy of surface patterns independent of any anisotropy of cell shape. For hepatocytes, the correlation between cell shape and apico-basal cell polarity is weak, for a discussion see [15].

Specifically, we radially project from the star-convex shape to a unit sphere concentric with the shape, retaining the nominal value of the original distribution, see S1C Appendix for additional details. To each cell with surface distribution *ρ*(**x**) of (apical) polarity proteins, we associate the projection *f*(**x**) of this distribution on the unit sphere $S^{2}$. Examples of this projection for an epithelial tubular cell from kidney tissue and a hepatocyte from liver tissue are shown in Fig 1F and 1G, respectively. Apical plasma membrane domains of these cells from kidney and liver tissue, respectively, were identified by staining fixed cells with anti-CD13 (Novus, cat NB100-64843, rat, 1/500) as reported previously [14, 15]; example raw images are shown in Fig S1A in S1B Appendix. The kidney cell exhibits clear vectorial polarity as reflected by a peak of the spherical power spectrum at the first mode. Such vectorial polarity is expected as kidney cells are regarded to belong to the vectorial cell polarity type also present in sheet-like epithelia [4]. The observation from a typical kidney cell from Fig 1F is confirmed for an ensemble of cells (*n* = 286); note that the relative magnitudes of spherical power modes exhibit a consistent pattern, whereas their absolute magnitude scales with the square of the projected area fraction of apical membrane.

**Fig 2:**
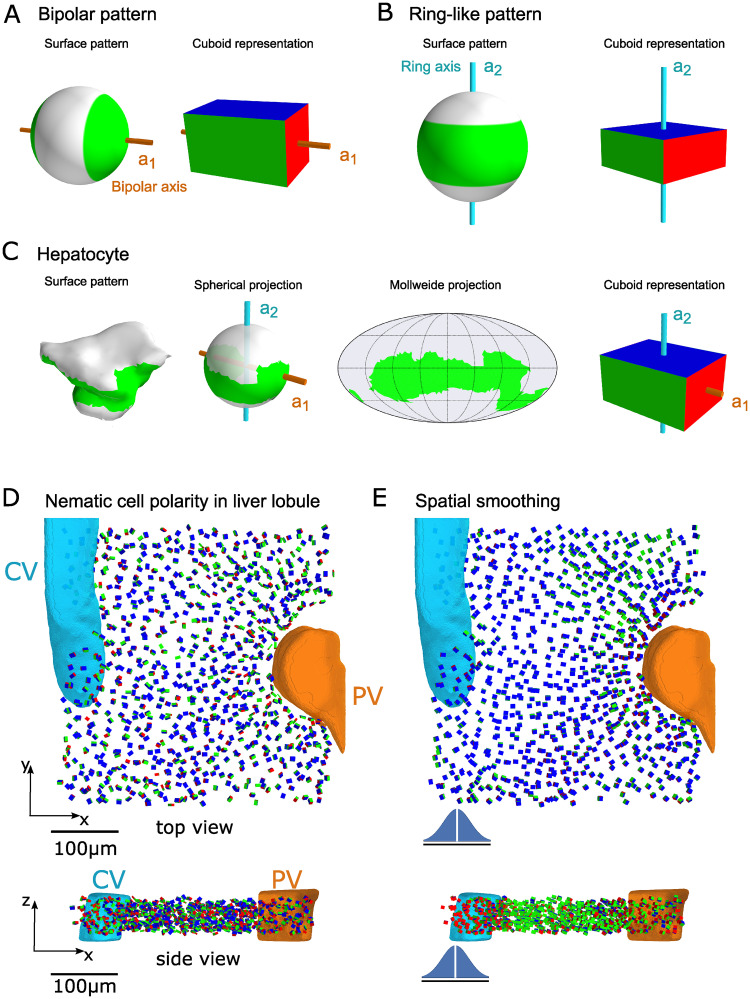
Spatial patterns of nematic cell polarity. We visualize surface distributions by cuboids that have the same moments of inertia tensor. Opposite faces of these cuboids are colored red, green, and blue, respectively, corresponding to the principal axes of inertia (ordered in increasing order). These principal axes of inertia correspond exactly to the principal axes of the nematic tensor ***A*** introduced in Eq (3) (ordered in decreasing order), such that the bipolar axis **a**_1_ (golden) is normal to the red face, and the ring axis **a**_2_ (cyan) is normal to the blue face of the cuboids (see S1E Appendix for details). (A) Idealized bipolar distribution. The *bipolar axis* **a**_1_ (golden) marks the principal axis of inertia of this surface distribution with largest eigenvalue *α*_1_, hence the *smallest* moment of inertia. We represent this bipolar surface distribution by a cuboid with same moments of inertia tensor. Thus, the bipolar axis corresponds to the red face (with smallest area). (B) Idealized ring-like distribution. The *ring axis* **a**_2_ (cyan) marks the principal axis of inertia with the smallest eigenvalue *α*_2_, hence the *largest* moment of inertia. In the cuboid representation of this ring-like distribution, the ring axis corresponds to the blue face of the cuboid (with largest area). (C) Bipolar and ring axis of a typical hepatocyte. From left to right: Apical membrane distribution for a typical hepatocyte, spherical projection, Mollweide projection, and cuboid representation. Shown are two distinguished principal axes of inertia **a**_1_ and **a**_2_, corresponding to the bipolar and ring nematic cell polarity axes, respectively. In the cuboid representation of the hepatocyte, the bipolar axis axis **a**_1_ corresponds again to the red face, whereas the ring axis **a**_2_ corresponds to the blue face. (D) For each hepatocyte in a tissue sample, the corresponding cuboid is plotted, revealing ordered patterns at the liver lobule level. (E) Orientational order becomes even more apparent after spatial averaging, which was performed using a Gaussian kernel with standard deviation of 20 *μm* and omitting the cell in the center (kernel sketched to scale, blue), see S1F Appendix for details. In panels (D) and (E), a central vein (CV, cyan) and a portal vein (PV, orange) are shown, which serve as landmarks within a liver lobule.

In contrast, for the hepatocyte, we find a dominant second mode, while the first mode is less pronounced. If spherical power spectra are averaged over a population of cells, we still find a dominant first mode for the case of kidney cells, see Fig 1F, and a pronounced second mode that exceeds the first mode in the case of hepatocytes, see Fig 1G. In fact, the first Fourier mode ||*F*_1_||^2^ was larger than the second mode ||*F*_2_||^2^ for 79% of the kidney cells, but only for 25% of the hepatocytes, see also Fig S1D, panel A in S1C Appendix. If we normalize Fourier modes by the squared mean ||*F*_0_||^2^ of the surface distribution, we observe a characteristic unimodular distribution of the normalized first mode ||*F*_*l*_||^2^/||*F*_0_||^2^ for kidney cells, and a characteristic unimodular distribution of the normalized second mode ||*F*_2_||^2^/||*F*_0_||^2^ for hepatocytes, see Fig S1D, panel B in S1C Appendix. Note that the large ensemble of cells analyzed here may contain segmentation errors. By manually examining hundreds of individual cases, we noticed that rare cases of potential segmentation errors seem to correspond to smaller values for the first and second mode of the Fourier spectrum. We thus expect that the ensemble-averaged spherical power spectra presented in Fig 1F and 1G represent a lower bound for the respective first and second mode, i.e., that the true values could be higher. The analysis of spherical power spectra of apical membrane distribution of kidney cells and hepatocytes (Fig 1F and 1G) highlights the structural difference between these two different cell types. While the concept of vectorial polarity well describes the polarity of kidney cells, hepatic cell polarity prompts for a description in terms of *nematic cell polarity* that focuses on the second Fourier mode. We introduce the nematic tensor ***A*** of the spherical distribution *f*(**x**),

$$\begin{array}{c} A=\frac{1}{2}\int_{S^{2}}\;d^{2}x\;f\left(x\right)(3\;x⊗x-1)\;, \end{array}$$ (3)

where 1 denotes the identity tensor with components $1_{ij}=\delta_{ij}$, *i*, *j* = 1, …, 3. The nematic tensor ***A*** encodes the same information as the second multipole *F*_2_(**x**). More generally, there is a formal link between the spherical modes of order *l* and the reduced Cartesian multipole moments [21], see also S1D Appendix. The nematic tensor ***A*** is closely related to a moments-of-inertia tensor, see S1E Appendix. We order the eigenvalues *α*_1_, *α*_2_, *α*_3_ of ***A*** such that *α*_1_ ≥ *α*_3_ ≥ *α*_2_ holds and denote the eigenvectors corresponding to *α*_1_, *α*_2_, *α*_3_ by **a**_1_, **a**_2_, **a**_3_ Motivated by Fig 2A and 2B, we will refer to **a**_2_ as the *ring axis* and **a**_1_ as the *bipolar axis*. Below, the axis **a**_2_ will represent an example of a *first* principal axis used to define co-orientational order parameters, while the axis **a**_1_ will represent the *second* principal axis. The numbering of axes **a**_2_ and **a**_1_ was chosen to be consistent with [15] (there *α*_*i*_ = *σ*_*i*_, *i* = 1, 2, 3).

### Cuboid representation of nematic cell polarity

To qualitatively assess putative spatial patterns of nematic cell polarity, we propose a visualization method in terms of equivalent cuboids, see Fig 2A–2C. Mathematically, the cuboid for a cell is uniquely determined by the condition that its traceless moments-of-inertia tensor should equal the traceless moments-of-inertia tensor of the spherical distribution *f*(**x**), see S1E Appendix. Briefly, the edges of the cuboid are parallel to the eigenvectors of the nematic tensor ***A*** associated to *f*(**x**), while the side-lengths of the cuboid depend on the eigenvalues of ***A***.

Fig 2A shows an idealized bipolar distribution and its equivalent cuboid. Here, the longest edge of the cuboid is parallel to the bipolar axis **a**_1_ of the surface distribution, while the two shorter axes have equal length. Similarly, for an idealized ring-like distribution, the shortest edge of the cuboid is parallel to the ring axis **a**_2_ of the surface distribution, while the two longest edges have equal length, see Fig 2B. We colored opposite faces of the cuboids in red, green, and blue, where red corresponds to the bipolar axis **a**_1_, and blue to the ring axis **a**_2_. Fig 2C shows the cuboid representation of a typical hepatocyte (with apical membrane distribution shown in green).

Next, we visualized spatial patterns of hepatic cell polarity within a liver lobule using the image segmentation of murine liver tissue from [15] (see Fig S1B in S1B Appendix for an example of the original raw image data). With the cuboidal representation introduced in Fig 2A–2C, we can draw an equivalent cuboid for each hepatocyte within a tissue section, thereby visualizing the biaxial nematic cell polarity of each hepatocyte with respect to its apical membrane distribution, see Fig 2D. In this figure panel, part of a liver lobule is shown with characteristic landmarks represented by the portal vein (orange) and the central vein (cyan). In top view, most of the polarity cuboids are faced with their blue side up (indicating an approximately parallel alignment of the ring axis **a**_2_ of hepatocyte polarity with the large veins; for the chosen tissue sample these are approximately parallel to the *z* axis). The visualization in terms of equivalent cuboids highlights the existence of a lobule-wide pattern of spatial order. The tissue-level alignment of nematic cell polarity becomes even more apparent when polarity fields are locally averaged to reduce fluctuations, see Fig 2D. Next, we will use order parameters from the theory of liquid crystals to quantify the observed spatial patterns of aligned cell polarity.

### Order parameters of nematic cell polarity

**Fig 3:**
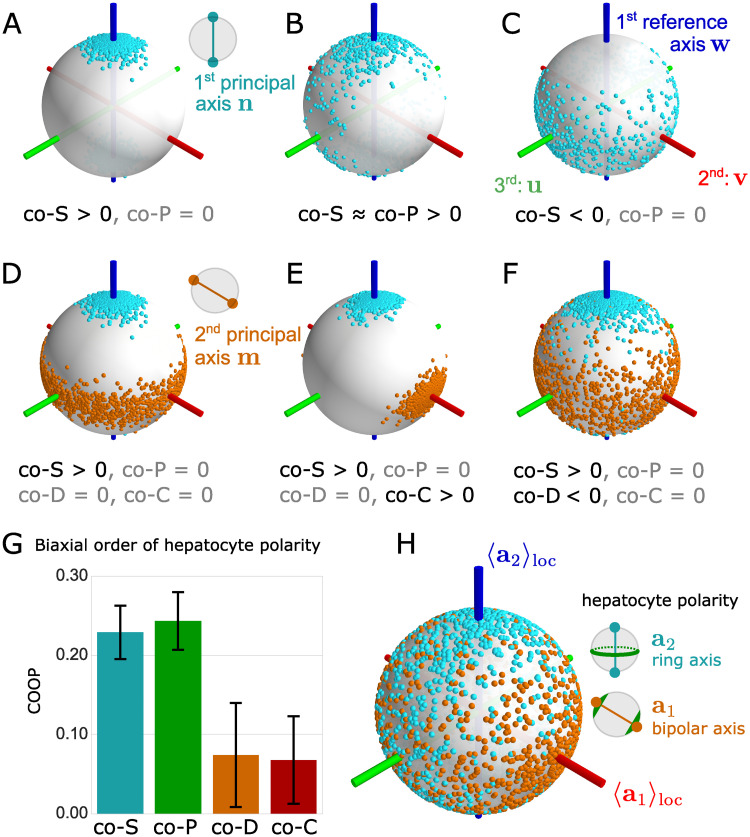
Four biaxial co-orientational order parameters applied to liver tissue. (A) Ensemble of first principal axes **n**, represented as pairs of antipodal points (cyan) on the unit sphere; the ensemble displays *prolate nematic order* with respect to the first reference axis **w** (blue). This type of order is characterized by a positive value of the co-orientational order parameter co−*S*, co−*S* > 0. First reference axis **w** (blue line), second reference axis **v** (red), third reference axis **u** (green). (B) Example of a *phase biaxial* distribution of first principal axes **n** (cyan dots) with nematic alignment towards the first reference axis **w** (blue) and strong anisotropic fluctuations biased towards the third reference axis **u** (green), corresponding to a positive value of the phase-biaxiality order parameter co−*P*, co−*P* > 0. (C) Example of *oblate nematic order* of **n** with respect to the first reference axis **w**, corresponding to co−*S* < 0. (D) Ensemble of tripods of principal axes **n**, **m**, **l** that displays *prolate nematic order* of the first principal axis **n** (cyan dots) with respect to the first reference axis **w** (blue), but no additional order of the second principal axis **m** (golden dots); third principal axis not shown. Since there is no additional order of **m**, we have co−*D* = co−*C* = 0. (E) Example of *molecular biaxial order* quantified by the co-orientational order parameter co−*C*. Here, the first principal axis **n** (cyan dots) displays prolate nematic order as in panel D, while the second principal axis **m** (golden dots) is additionally biased towards the second reference axis **v** (red), corresponding to co−*C* > 0. (F) A second type of molecular biaxial order is measured by the co-orientational order parameter co−*D*. Here, the first principal axis **n** (cyan dots) exhibits nematic order with respect to the first reference axis **w** (blue). Fluctuations of the second principal axis **m** (golden dots) are also biased towards **w**, corresponding to co−*D* < 0. (G) Co-orientational order parameters quantify biaxial order of hepatocytes in liver tissue (mean±s.d., *n* = 12 tissue samples). The local reference system was chosen as a local average with punctured Gaussian kernel, see text for details. (H) Spherical distribution of apical ring axis **a**_2_ (antipodal pairs of cyan dots) and apical bipolar axis **a**_1_ (golden dots) of hepatocyte cell polarity relative to the reference axes **w** = 〈**a**_2_〉_loc_ (blue), **v** = 〈**a**_1_〉_loc_ (red), **u** = **v** × **w** (green), illustrating the quantitative analysis in panel G.

We can quantify orientational order of nematic cell polarity within a tissue in terms of orientational order parameters (OOP), *S*, *P*, *D*, *C*, see Fig 3. These order parameters were originally developed for the study of biaxial order in liquid crystals [22, 23]. We briefly review their definition and provide illustrative examples to convey their geometric meaning. A short computer program accompanying this work allows to interactively explore prototypical examples.

Before we present the formal definition of *S*, *P*, *D*, *C* in Eq (7), we want to motivate the different roles played by the four OOP. For an ensemble of uniaxial objects, each characterized by a single principal axis, *S* quantifies how well the ensemble of these principal axes are aligned to a common mean direction. The mean direction of this nematic alignment defines one of several reference axes introduced below. In the theory of simple liquid crystals, *S* is the most widely used OOP (and probably the most important one). Like *S*, the OOP *P* is also defined already for an ensemble of uniaxial objects (with a single principal axis each). While *S* characterizes the strength of nematic alignment of the single principal axis in the ensemble, *P* characterizes anisotropic fluctuations of this principal axis, i.e., *P* becomes non-zero if the deviations of the principal axes from the mean direction are skewed in a particular direction. The direction of anisotropic fluctuations defines a second reference axis.

The two other OOP, *D* and *C*, are only defined for biaxial objects that are characterized by *two* principal axes (a third principal axis can be deduced from the two). The OOP *C* characterizes nematic alignment of the second principal axis towards a second reference axis. Finally, *D* quantifies an ‘unexpected relation’ between the fluctuations of the first and second principal axis, respectively, with respect to their alignment to a first reference axis of mean alignment. For a maximum-entropy distribution of orientations, constrained to specific numerical values of *S*, *P*, *C*, the OOP *D* would be zero.

In liquid crystals, built up by an ensemble of anisotropic molecules, a non-zero value of *S* can arise from the interactions of uniaxial molecules (e.g., molecules approximated as rods) with a uniaxial external field. Here, the axis of the external field sets the direction of mean alignment, i.e., the first reference axis. A non-zero value of *P*, however, additionally requires either a second external field, orthogonal to the first one, or boundary conditions that break rotational symmetry for rotations around the first reference axis. Non-zero values of *D* and *C* obviously require objects that are intrinsically biaxial, i.e., that do not possess rotational symmetry around their first principal axis. While a non-zero value of *D* can result already from interactions of biaxial objects with an external uniaxial field, a non-zero value of *C* requires either an external field that is biaxial, boundary conditions that break uniaxial symmetry, or, possibly, biaxial inter-molecular interactions.

Generally, the order parameters *S* and *P* characterize order in an ensemble of uniaxial objects characterized by just a single nematic axis. In this case, *S* and *P* depend only on the second spherical mode of the distribution of axes and can be computed in terms of the coefficients $f_{l}^{m}$ of an expansion of this distribution into spherical harmonics, provided a special choice of coordinate system is made, see S1D Appendix for details.

We now proceed to the formal definition of OOP, which will later be generalized to the case of co-orientational order parameters (COOP) and applied to quantitatively characterize design principles of liver tissue. We consider an ensemble of cuboids, each characterized by a tripod of principal axes, which we characterize by orthonormal vectors **n**, **m**, **l**. These unit vectors are only defined up to sign, thus any meaningful physical quantity should be invariant under sign flips **n** → −**n**, **m** → −**m**, **l** → −**l**. Formally, each cuboid is said to possess so-called *D*_2*h*_-symmetry, i.e., it is invariant under line reflections at its principal axes. We distinguish a *first principal axis* **n**, as well as a *second principal axis* **m**, and a *third principal axis* **l**. It is convenient to introduce, for each cuboid, two traceless tensors ***Q*** and ***B*** that characterize its tripod of axes [23, 24]

$$\begin{array}{c} Q=\frac{1}{2}(3\;n⊗n-1)\;,\;B=\frac{3}{2}(l⊗l-m⊗m)\;. \end{array}$$ (4)

Here, 1 is the identity tensor and ⊗ denotes the outer product.

If the principal axes **n**, **m**, **l** of each tripod are given by the normalized eigenvectors of a nematic tensor ***A*** (e.g., the nematic tensor of a projected surface distribution *f*(**x**) of membrane proteins), we can recover ***A*** as linear superposition of the two tensors ***Q*** and ***B***, see S1J Appendix. The mathematical advantage of the traceless tensors ***Q*** and ***B*** is that they conveniently allow to compute ensemble averages, 〈***Q***〉 and 〈***B***〉. The eigenvalues of these averaged tensors provide important invariants of orientational order

$$\begin{array}{cc} R_{Q}^{T}\;\left\langle Q\right\rangle \;R_{Q} & =(\begin{array}{ccc} -\frac{1}{2}\left(S-P\right) & 0 & 0\\ 0 & -\frac{1}{2}\left(S+P\right) & 0\\ 0 & 0 & S \end{array})\;, \end{array}$$ (5)

$$\begin{array}{cc} R_{B}^{T}\;\left\langle B\right\rangle \;R_{B} & =(\begin{array}{ccc} -\frac{1}{2}\left(D-3\;C\right) & 0 & 0\\ 0 & -\frac{1}{2}\left(D+3\;C\right) & 0\\ 0 & 0 & D \end{array})\;. \end{array}$$ (6)

Here, ***R***_*Q*_ and ***R***_*B*_ are rotation matrices that diagonalize 〈***Q***〉 and 〈***B***〉, respectively.

In principle, the rotation matrices ***R***_*Q*_ and ***R***_*B*_ might be different. However, for important special cases, e.g., ensembles of biaxial molecules interacting with simple external fields, both rotation matrices can be chosen equal, ***R*** = ***R***_*Q*_ = ***R***_*B*_. In this case, the ensemble-averaged tensors 〈***Q***〉 and 〈***B***〉 possess the same eigenvector basis, given by three mutually orthogonal unit vectors **w**, **v**, **u**. The physical meaning of **w**, **v**, **u** is that these vectors define mutually orthogonal symmetry axes, such that the statistics of the ensemble of cuboids is invariant under line reflections at these axes. The ensemble of cuboids is said to possess *D*_2*h*_-*symmetry* in this case. In the case of *D*_2*h*_-symmetry, the common eigenvectors **w**, **v**, **u** of 〈***Q***〉 and 〈***B***〉 define a *director frame of reference axes* of the ensemble. Note ***R***_*Q*_ = ***R***_*B*_ = [**u**, **v**, **w**] (with column-vectors **u**, **v**, **w**).

Using Eqs (5) and (6), we can rewrite *S*, *P*, *D*, *C* as averaged direction cosines [23]

$$\begin{array}{cc} S & =\frac{1}{2}\langle 3{\left(n^{\left(i\right)}\cdot w\right)}^{2}-1\rangle \;,\\ P & =\frac{3}{2}\langle {\left(n^{\left(i\right)}\cdot u\right)}^{2}-{\left(n^{\left(i\right)}\cdot v\right)}^{2}\rangle \;,\\ D & =\frac{3}{2}\langle {\left(l^{\left(i\right)}\cdot w\right)}^{2}-{\left(m^{\left(i\right)}\cdot w\right)}^{2}\rangle \;,\\ C & =\frac{1}{2}\langle {\left(l^{\left(i\right)}\cdot u\right)}^{2}-{\left(l^{\left(i\right)}\cdot v\right)}^{2}+{\left(m^{\left(i\right)}\cdot v\right)}^{2}-{\left(m^{\left(i\right)}\cdot u\right)}^{2}\rangle \;, \end{array}$$ (7)

where 〈⋅〉 averages over an ensemble of nematic axes **n**^(*i*)^, **m**^(*i*)^, **l**^(*i*)^ indexed by *i*, using a fixed tripod of reference axes **w**, **v**, **u**.

In Eq (7), there is ambiguity regarding the ordering of the various axes, **n**, **m**, **l**, and **w**, **v**, **u**. For our specific case, the mapping from the nematic cell polarity axes **a**_1_, **a**_2_, **a**_3_ to the principal axes **n**, **m**, **l** is only defined up to a constant permutation

$$\begin{array}{c} n={\text{a}}_{\pi (2)}\;,\;m={\text{a}}_{\pi (1)}\;,\;l={\text{a}}_{\pi (3)},\;\pi \in S_{3}\;, \end{array}$$ (8)

where *S*_3_ denotes the group of all permutations of the indices (1, 2, 3). Note that the same permutation *π* must be used for all tripods of nematic axes ${\text{a}}_{1}^{\left(i\right)}$, ${\text{a}}_{2}^{\left(i\right)}$, ${\text{a}}_{3}^{\left(i\right)}$ of the ensemble. Similarly, if **e**_1_, **e**_2_, **e**_3_ denote the common eigenvectors of 〈***Q***〉 and 〈***B***〉 (with some fixed ordering), we distinguish a *first reference axis* **w** = **e**_*ρ*(2)_ from a *second reference axis* **v** = **e**_*ρ*(1)_, and a *third reference axis* **u** = **e**_*ρ*(3)_, where *ρ* ∈ *S*_3_ denotes a permutation of reference axes. (The axes **w**, **v**, **u** are also called director axes [23]).

We distinguish two different choices for *π* and *ρ*, which give rise to orientational order parameters (OOP) as commonly used in the theory of liquid crystals [23], and co-orientational order parameters (COOP) introduced here.

A common choice, put forward, e.g., by Zannoni et al. [23] in the field of biaxial nematics, is to chose the permutations *π* and *ρ* of principal and reference axes such that

$$\begin{array}{c} |S|\;\text{is}\;\text{maximal,}\;P\geq 0,\;\text{and}\;C\geq 0. \end{array}$$ (9)

The condition Eq (9) specifies an ordering of both principal and reference axes (S1I Appendix discusses how a permutation of axes affects the OOP). The tensor 〈***Q***〉 and the scalar order parameters *S* and *P* quantify alignment of the first principal axis **n**, whereas the tensor 〈***B***〉 and the scalar order parameters *D* and *C* quantify the alignment of the second and third principal axes, **m** and **l**. We will refer to the values of *S*, *P*, *D*, *C* corresponding to the ordering of nematic axes specified by Eq (9) as *order parameters* (OOP) without further specification. Note that different normalization conventions for OOP are in use, an overview can be found in [21]. While OOP are always well-defined, they have a crucial disadvantage: OOP may change discontinuously if system parameters are smoothly varied due to abrupt changes of either *π* or *ρ*, see S1J Appendix.

We propose an alternative choice, where the ordering of axes is directly determined by the properties of a nematic tensor ***A***. In the case of surface distributions on a sphere considered here, we take **n** to point in the direction of the ring axis **a**_2_, and **m** to point in the direction of the bipolar axis **a**_1_.

We consider the general case, where for each nematic tensor ***A***^(*i*)^ from an ensemble of tensors indexed by *i*, we additionally have a second nematic tensor ***E***^(*i*)^ for each *i*. Below, we discuss two natural cases of such reference tensors. Let *ε*_1_, *ε*_2_, *ε*_3_ be the eigenvalues of one of the ***E***^(*i*)^, ordered such that *ε*_1_ ≥ *ε*_3_ ≥ *ε*_2_, and **e**_1_, **e**_2_, **e**_3_ be the corresponding (normalized) eigenvectors. We introduce a tripod of reference axes for each index *i* as **w**^(*i*)^ = **e**_2_, **v**^(*i*)^ = **e**_1_, **u**^(*i*)^ = **e**_3_, i.e., we chose *ρ* as the identify permutation, *ρ* = id. We define *co-orientational order parameters* (COOP) by generalizing Eq (7) to this general case, where the reference axes **u**^(*i*)^, **v**^(*i*)^, **w**^(*i*)^ are derived from a set of reference tensors ***E***^(*i*)^

$$\begin{array}{cc} \text{co}-S & =\frac{1}{2}\langle 3{\left(n^{\left(i\right)}\cdot w^{\left(i\right)}\right)}^{2}-1\rangle \;,\\ \text{co}-P & =\frac{3}{2}\langle {\left(n^{\left(i\right)}\cdot u^{\left(i\right)}\right)}^{2}-{\left(n^{\left(i\right)}\cdot v^{\left(i\right)}\right)}^{2}\rangle \;,\\ \text{co}-D & =\frac{3}{2}\langle {\left(l^{\left(i\right)}\cdot w^{\left(i\right)}\right)}^{2}-{\left(m^{\left(i\right)}\cdot w^{\left(i\right)}\right)}^{2}\rangle \;,\\ \text{co}-C & =\frac{1}{2}\langle {\left(l^{\left(i\right)}\cdot u^{\left(i\right)}\right)}^{2}-{\left(l^{\left(i\right)}\cdot v^{\left(i\right)}\right)}^{2}+{\left(m^{\left(i\right)}\cdot v^{\left(i\right)}\right)}^{2}-{\left(m^{\left(i\right)}\cdot u^{\left(i\right)}\right)}^{2}\rangle \;, \end{array}$$ (10)

where 〈⋅〉 averages over the ensemble of pairs of tripods indexed by *i*. We propose a scheme to compute reference tensors ***E***^(*i*)^ for the important case, where the tensors ***A***^(*i*)^ = ***A***(**x**^(*i*)^) depend on spatial position **x**^(*i*)^. For each position **x**^(*i*)^, we define ***E***^(*i*)^ = 〈***A***(**x**^(*i*)^)〉_loc_ using a local average with a “punctured” three-dimensional Gaussian kernel centered at **x**^(*i*)^ (excluding the tensor ***A***^(*i*)^ at the central position **x**^(*i*)^), see S1F Appendix for details. The definition of reference axes in terms of local averages provides a robust definition of reference frame if the direction of nematic order varies as function of spatial position. Indeed, the visualization of nematic cell polarity in liver tissue indicates a curved director field of nematic cell polarity on the lobule-level, see Fig 2D and 2E.

Below, we additionally consider a variation of this theme, where the tripod of reference axes **n**, **m**, **l** is not given by a local average, but by a second set of biaxial objects (namely the local anisotropy of the sinusoid transport network in the liver).

The most important difference between the traditional definition of the OOP, *S*, *P*, *D*, *C*, and our definition of COOP, co−*S*, co−*P*, co−*D*, co−*C*, is that the permutation *π* of principal axes, and *ρ* of reference axes is determined by the orientational order of the ensemble itself for OOP, but prescribed by the eigenvalues of a nematic tensor for COOP. This apparently small change in the mathematical definition, i.e., fixing the order of reference axes, renders co-orientational order parameters (COOP) a robust metric that is applicable also in the case of curved director fields, where classical order parameters (OOP) may change discontinuously.

Below, we will apply these biaxial co-orientational order parameters to quantify the alignment of nematic cell polarity of hepatocytes in liver tissue.

#### Geometric meaning of order parameters

We illustrate the geometric meaning of the co-orientational order parameters (COOP) co−*S*, co−*P*, co−*D*, co−*C* introduced in Eq (10) see Fig 3. In short, these COOP characterize the alignment of nematic principal axes **n**, **m**, **l** relative to a set of (mutually perpendicular) reference axes **w**, **v**, **u**. These COOP generalize the known orientational order parameters *S*, *P*, *D*, *C*, see Eq (7), for which the reference axes are directly inferred from the ensemble of principal axes itself.

When co−*S* > 0 and all other co-orientational order parameters vanish, as in panel 3A, the ensemble is said to possess *uniaxial prolate order* (also called cluster-type order [25]). Such uniaxial orderings are axially symmetric around their first reference axis **w**. If fluctuations of the first principal axis **n** are anisotropic, as shown in panel 3B, the ensemble is said to possess *phase-biaxial order*. Such anisotropic fluctuations are quantified by the magnitude of the co-orientational order parameter co−*P*. In panel 3C, an axially-symmetric distribution with co−*S* < 0 is shown, termed *uniaxial oblate* order, where the first principal axis **n** scatters close to the ‘equator’ (with north-pole south-pole axis set by **w**). Uniaxial oblate order is sometimes also called *girdle order* [25].

So far, we only examined the distribution of the first principal axis **n**, which is quantified by the co-orientational order parameters *S* and *P*. We now turn to the full description of biaxial nematic order, characterizing the distribution of a tripod of axes, **n**, **m**, **l**. In panels 3D, 3E, and 3F, we show examples of an additional ordering of a second principal axis **m**, which are quantified by the other two co-orientational order parameters co−*D* and co−*C*. We illustrate distributions of the second principal axis **m** by antipodal pairs of golden points on the sphere. Panel D shows the reference case of an uniaxial prolate distribution as in panel 3A. In absence of any additional ordering, the axis **m** displays uniaxial oblate order, as it is must be perpendicular to the first principal axis **n**. Comparison of Fig 3A and 3D demonstrates that the type of order (prolate or oblate) crucially depends on which axis is chosen as the first principal axis.

We now consider the case of an additional ordering of the second principal axis **m**. In panel 3E, **m** aligns towards the second reference axis **v** (red). This alignment of **m** breaks axial symmetry around **w** for the second principal axis **m** (golden), but not for the first principal axis **n** (cyan). Correspondingly, the co-orientational order parameter co−*P* describing the phase biaxiality of the first principal axis **n** remains zero, but the *molecular biaxiality parameter* co−*C* becomes nonzero. The parameter co−*C* thus describes the deviation from axial symmetry with respect to the first reference axis **w** of the ensemble of second principal axes **m**. In contrast, both the first and second principal axis, **n** and **m**, compete for the same reference axis **w** in panel F. Correspondingly, their respective distributions remain axially symmetric around **w**. In this case, both co−*P* and co−*C* are zero, yet the *molecular ordering parameter* co−*D* is non-zero.

### Application to liver tissue

We now apply the framework of biaxial order parameters to quantify lobule-level patterns of nematic cell polarity in mouse liver tissue. We first examine the co-orientational order of the apical nematic polarity of hepatocytes with respect to its own local average. As detailed in the preceding section, we compare the nematic polarity axes of each individual hepatocyte (introduced in Fig 2C) with a local reference frame, given by a local average of the tensors ***A*** with a punctured Gaussian kernel (illustrated in Fig 2E). This provides reference axes **w** = 〈**a**_2_〉_loc_, **v** = 〈**a**_1_〉_loc_, and **u** = **v** × **w** at each hepatocyte position. (Mathematically, 〈⋅〉_loc_ is defined using nematic tensors, see S1F Appendix for details). We choose the first principal axis **n** to point in the direction of the ring axis **a**_2_, and the second principal axis **m** to point in the direction of the bipolar axis **a**_1_.

This choice uniquely specifies the four co-orientational order parameters, see Fig 3G. As additional illustration, we show the distribution of nematic cell polarity axes relative to its local reference system, see Fig 3H. We find that the ring axis **a**_2_ (cyan dots) is clustered around the first reference axis **w** = 〈**a**_2_〉_loc_. Correspondingly, the scalar order parameter co−*S* of uniaxial nematic order is larger than zero. Additionally, we find a statistically significant phase biaxiality with co−*P* > 0, revealing that fluctuations of the ring axis **n** = **a**_2_ are biased away from the average bipolar axis **v** = 〈**a**_1_〉_loc_. This phase biaxiality is also visible in the distribution plot on the sphere in Fig 3H. The second principal axis **m** (bipolar axis **a**_1_, golden dots) also exhibits a weak ordering with a bias towards **v** (averaged bipolar axis 〈**a**_1_〉_loc_, red) and away from **w**, reflected by positive values of co−*C* and co−*D*, respectively. Thus, using co-orientational order parameters that compare nematic axes with a local average (omitting the central cell), we can rigorously assess biaxial order even in the presence of curved director fields. Moreover, these COOP allow to characterize phenotypes quantitatively. Fig S1E in S1G Appendix reports COOP for Integrin-*β*1 knockdown mice [15], demonstrating that COOP of hepatocyte polarity are substantially reduced in comparison to normal conditions. In these knockdown mice, communication between sinusoidal cells and hepatocytes is impaired due to selective siRNA silencing of a transmembrane ECM receptor in hepatocytes.

### Co-alignment of nematic cell polarity and local anisotropy of blood transport network

**Fig 4:**
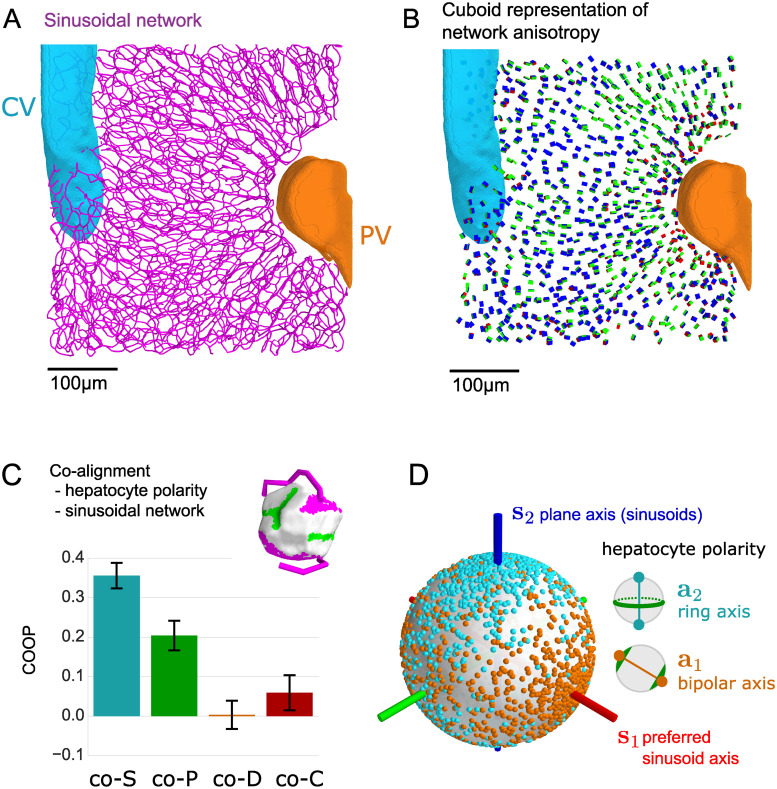
Biaxial order of sinusoidal network correlates with nematic cell polarity. (A) Central lines of the sinusoidal network in the liver lobule (same section of mouse liver tissue as in Fig 3; central vein: cyan, portal vein: orange). (B) The local anisotropy of the sinusoidal network is visualized by cuboids with equivalent moments-of-inertia tensor (using spherical regions of interest centered at each hepatocyte position of 20 *μm* radius). (C) Co-orientational order between apical nematic cell polarity and local anisotropy of the sinusoidal network, quantified in terms of the co-orientational order parameters introduced in Eq (10), where the principal axes are given by the axes of hepatic cell polarity for individual hepatocytes (**n** = **a**_2_, **m** = **a**_1_, **l** = **a**_3_), and the reference axes are given by the axes of the local anisotropy of the sinusoidal network (**w** = **s**_2_, **v** = **s**_1_, **u** = **s**_3_); (mean±s.d., *n* = 12 tissue samples). We find co−*S* > 0, showing that the ring axis **a**_2_ of hepatic cell polarity is preferentially aligned parallel to the plane axis **s**_2_ of the sinusoidal network, i.e., the ring axis is normal to the local layered organization of the sinusoidal network. Fluctuations of the ring axis are biased away from the preferred sinusoid axis **s**_1_, corresponding to co−*P* > 0. Note that **s**_1_ is approximately aligned with the direction of blood flow [15], while **s**_2_ is approximately parallel to the large veins. The COOP co−*D* and co−*C* characterize any additional alignment of the bipolar axis **a**_1_ of hepatic cell polarity; we find that co−*D* and co−*C* are not significantly different from zero. The inset shows a typical hepatocyte with apical membrane (green), basal membrane (magenta), and the sinusoidal network in a spherical region of interest of radius 20 *μm* centered at the position of the hepatocyte (magenta). While the apical membrane defines the cell polarity axes, the local sinusoidal network defines local reference axes, used in the definition of the COOP. (D) Spherical distribution of the apical ring axis of hepatocyte polarity **a**_2_ (represented as antipodal pairs of cyan dots on the unit sphere) and apical bipolar axis **a**_1_ (golden dots), relative to the reference frame of local sinusoidal network anisotropy, represented by the local preferred sinusoid axis **s**_1_ (red) and the plane axis **s**_2_ of the sinusoidal network that characterized its layered organization (blue).

We can analyze nematic order of cell polarity not only within an ensemble of cells, but also quantify the mutual alignment between cell polarity and auxiliary anisotropic structures such as transport networks. As example, we analyze co-orientational order between apical nematic cell polarity of hepatocytes, and the local anisotropy of the blood-transporting sinusoidal network [15, 26]. Sinusoids are specialized blood vessels forming a network within the liver lobule [10]. Fig 4A shows a central-line representation of the sinusoidal network.

We determine the local anisotropy of the sinusoidal network in the vicinity of each hepatocyte. Specifically, if **d**_*k*_ are unit vectors parallel to straight network segments, **x**_*k*_ their midpoint positions and *l*_*k*_ their respective lengths, we define nematic tensors ***S*** at each hepatocyte position **x**^(*i*)^

$$\begin{array}{c} S\left(x^{\left(i\right)}\right)=\sum_{k}w\left(x_{k}-x^{\left(i\right)}\right)\;l_{k}(d_{k}⊗d_{k}-\frac{1}{3}1)\;. \end{array}$$ (11)

Here, *w*(**x**) is a weighting function normalized as ∑_*k*_ *w*(**x**_*k*_ − **x**^(*i*)^)*l*_*k*_ = 1. We choose *w*(**x**) as a binary cutoff with fixed radius of 20 *μ*m around the center of each hepatocyte. The geometric meaning of ***S*** can be understood as follows: The eigenvector **s**_1_, corresponding to the largest eigenvalue, characterizes the direction of preferred sinusoid orientation and will be referred to as *preferred axis*. The eigenvector **s**_2_, corresponding to the smallest eigenvalue, defines the normal to a plane in which sinusoids orientations are preferentially distributed, and will be referred to as *plane axis* in the following. The biaxial anisotropy of the sinusoidal network with a distinguished plane axis is indicative of a local layered order, where **s**_2_ represents the normal vector of a stack of parallel layers, which characterizes this layered order. Approximately, **s**_2_ is parallel to both the centerline of the portal vein, and the centerline of the central vein. Fig 4B shows the spatial distribution of these nematic axes, using cuboids with equivalent moments of inertia. The pattern of network anisotropy is similar to the averaged pattern of apical cell polarity: The preferred sinusoid axis **s**_1_ represented by the red faces of the cuboids is oriented approximately along the mean direction of blood flow between the portal and central vein (and thus almost not visible in Fig 4B), reported already in [15]. The plane axis **s**_2_ represented by the blue faces of the cuboids is preferentially aligned parallel to the large veins, representing a local layered organization of this network. This anisotropy of the sinusoidal network with a preferred nematic axis and a local layered organization defines a local reference frame, against which we can compare hepatocyte cell polarity. Fig 4C shows the four co-orientational order parameters between apical nematic cell polarity and local anisotropy of the sinusoidal network. We find that the ring axis **a**_2_ of apical cell polarity is approximately aligned with the plane axis **s**_2_ of the local sinusoid anisotropy, corresponding to co−*S* > 0. In an idealized picture, the rings of apical cell membrane surrounding hepatocytes would be parallel to the parallel layers of the sinusoidal network. The COOP co−*P* > 0 shows that fluctuations of the ring axis **a**_2_ around **s**_2_ are not random, but are biased towards the third reference axis **s**_3_ = **s**_1_ × **s**_2_ of the sinusoidal network, and thus away from the preferred axis **s**_1_ of nematic alignment of this network. In physical terms, this implies that liquid-crystal order in liver tissue is truly biaxial (exhibiting so-called phase-biaxial order). The other co-orientational order parameters co−*D* and co−*C* are close to zero, i.e., we do not find a particular ordering of the bipolar cell polarity axis **a**_1_ relative to the anisotropic sinusoidal network.

The COOP provide insight about the mutual arrangement of the two transport networks in liver tissue, the sinusoidal network and the bile canaliculi network. The apical membrane domains of hepatocytes indicate the contact surfaces between hepatocytes to the bile canaliculi network. The co-alignment of the ring axis **a**_2_ of hepatocytes and the plane axis **s**_2_ of the sinusoidal network quantified by co−*S* > 0 suggests a sandwich architecture of these two intertwined networks with alternating layers enriched in either bile canaliculi or sinusoidal network. The observed anisotropic fluctuations of **a**_2_ quantified by co−*P* > 0 could reflect an effective mutual repulsion between the sinusoidal and the bile canaliculi network.

The co-orientational order is also visualized as a spherical distribution plot in Fig 4D, highlighting the biaxial co-alignment between hepatocyte polarity and the anisotropy of the sinusoidal network in liver tissue.

### Minimal model for co-orientational order

We present a minimal interaction model that can quantitatively reproduce the co-alignment between hepatocyte cell polarity and the biaxially anisotropic sinusoidal network. If we account only for the ring axis **a**_2_ of hepatocytes, the leading order term of an effective interaction energy is dictated by symmetry and reads

$$\begin{array}{c} H=-\lambda \;\alpha_{2}\;({\text{a}}_{2}⊗{\text{a}}_{2}):S\;, \end{array}$$ (12)

where *α*_2_ ≈ −0.19 is the average eigenvalue corresponding to the ring axis **a**_2_ of the tensors ***A*** of nematic hepatocyte polarity, see Eq (3), and ***S*** is a nematic tensor that characterizes the local anisotropy of the sinusoidal network, see Eq (11) and S1H Appendix. Further, (**a**_2_ ⊗ **a**_2_):***S*** denotes the contraction of the two tensors ***A***′ = **a**_2_ ⊗ **a**_2_ and ***S***, i.e., $A^{\prime }:S=\sum_{i,j=1}^{3}A_{ij}^{\prime }S_{ij}=\sum_{i,j=1}^{3}a_{2,i}a_{2,j}S_{ij}$, where *a*_2,*i*_, *a*_2,*j*_ denote the components of the vector **a**_2_. Thus, we treat the hepatocytes as uniaxial objects, whereas we retain the biaxial anisotropy of the sinusoid network. The choice of Eq (12) is motivated by a general Landau theory of liquid crystals, see [23, 24]. We calculate the order parameters of an ensemble of axes according to the Boltzmann distribution following this Hamiltonian, using inverse sampling. The control parameter λ is measured in units of an effective temperature that mimics dynamic processes that reduce spatial order [27]. We emphasize that we do not consider liver tissue to represent a thermodynamic equilibrium, despite our use of Eq (12). Instead, Eq (12) represents a phenomenological model that addresses a competition between dynamic processes that either generate or reduce spatial order, respectively.

**Fig 5:**
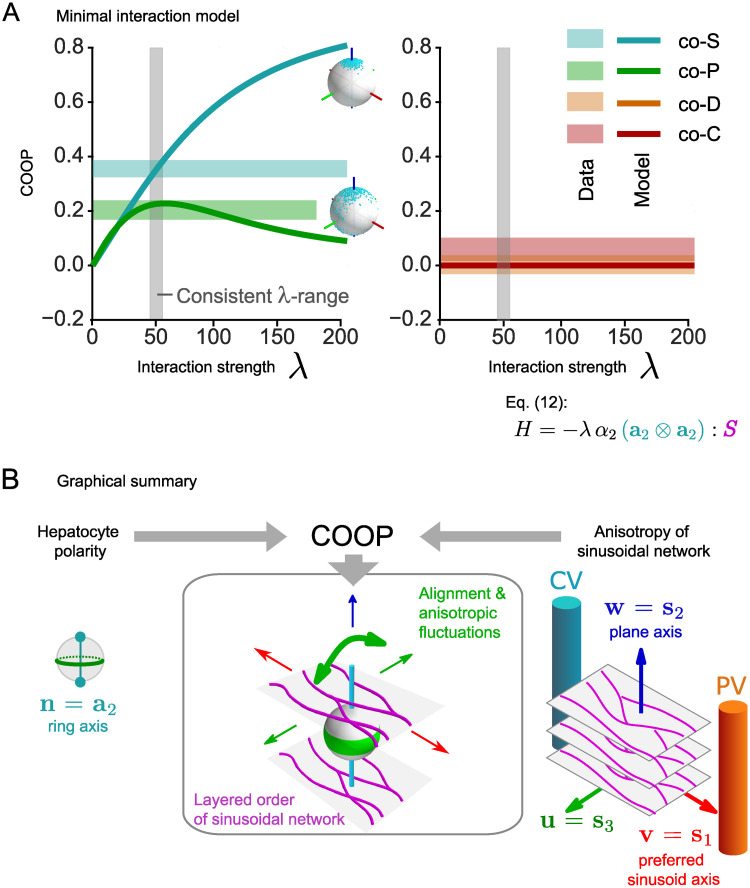
Minimal interaction model reproduces biaxial order parameters for hepatocyte/sinusoid co-alignment. (A) Simulated co-orientational order parameters (COOP) between nematic cell polarity axes and local anisotropy of the sinusoidal network as function of the dimensionless interaction strength λ (solid lines) for the minimal interaction model given in Eq (12). The color code for co−*S*, co−*P*, co−*D*, co−*C* corresponds to that of Figs 3 and 4; the insets reshow the spherical distribution plots from Fig 3A and 3B for the cases co−*S* > 0 and co−*P* > 0. Shaded regions indicate mean±s.d. of experimental values from Fig 4C. The range of λ for which all four order parameters agree in simulation and experiment is highlighted in gray. Note that the model prediction for co−*C* (golden solid line) coincides with the prediction for co−*D* (brown solid line) and is thus not visible. (B) Graphical summary of co-orientational order between hepatocyte polarity and the local anisotropy of the sinusoidal network. The anisotropy of the sinusoidal network defines local reference axes: a preferred sinusoid axis **s**_1_ (red, approximately parallel to the mean direction of blood flow), a plane axis **s**_2_ (blue, characterizing a layered organization of the sinusoidal network), and a third axis **s**_3_ = **s**_1_ × **s**_2_ perpendicular to the other two axes (green). Nematic cell polarity of hepatocytes defines two nematic axes for each cell: a ring axis **a**_2_ and a bipolar axis **a**_1_, of which the ring axis co-aligns with the sinusoidal network. The ring axis **a**_2_ (cyan) aligns parallel to the plane axis **s**_2_ of the local sinusoidal network. This reflects a sandwich architecture of the two intertwined networks of bile canaliculi and sinusoids, with alternating layers enriched in apical membrane of hepatocytes, which represent contact surfaces to the bile canaliculi network, and endothelial cells forming the sinusoidal network, which is quantified by co−*S* > 0. Fluctuations of the ring axis **a**_2_ are not random, but are biased towards the third reference axis **s**_3_ of the sinusoidal network (green), and thus away from the preferred axis **s**_1_ (red), as quantified by co−*P* > 0. These anisotropic fluctuations supposedly reflect an effective mutual repulsion between the sinusoidal and the bile canaliculi network. For the bipolar axis **a**_1_ of hepatocyte polarity, we do not find (unexpected) co-orientational order relative to the sinusoidal network, corresponding to co−*D* ≈ 0 and co−*C* ≈ 0.

Fig 5A displays computed COOP, together with the regions of order parameters found for the experimental data of liver tissue. We find a range of values of the effective interaction strength λ (shaded gray in Fig 5A), where the minimal model adequately accounts for the experimental observed values of the co-orientational order parameters. Thus, the interaction of the ring axis **a**_2_ of hepatocytes with the local anisotropy of the sinusoidal network is sufficient to account for the observed biaxial co-orientation. Intriguingly, alternative models assuming either an interaction between ***S*** and the bipolar axis **a**_1_, or the full tensor ***A***, did not reproduce the observed co-orientational order, see S1H Appendix.

This finding suggests the cartoon picture of sinusoid-hepatocyte co-alignment in liver tissue shown in Fig 5B. We propose that the ring axis **a**_2_ of hepatocytes preferentially aligns parallel to the plane axis **s**_2_ of the local sinusoidal network. Fluctuations of **a**_2_ break axial symmetry and are biased away from the preferred axis **s**_1_ of the sinusoidal network.

## Discussion

We presented a general method to identify and quantify different types of cell polarity, based on a multipole decomposition of surface patterns. Using our method, we classify cell polarity as vectorial polarity, nematic polarity, or higher-order type. For this, surface distributions of apical cell membrane of star-convex cells were projected on a unit sphere. In principle, our method could be extended to cells that are not star-convex or for which image segmentation is not available, provided a midpoint of each cell can be defined (e.g. by tracking the nucleus). In this case, a three-dimensional distribution of fluorescence intensity in a spherical neighborhood of this midpoint could be projected on the unit sphere concentric with the midpoint, without prior image segmentation.

We applied our method to three-dimensional reconstructions of epithelial tissue cells, and the distribution of apical membrane markers on their surface [14]. We confirm that kidney cells predominantly display vectorial cell polarity. In contrast, hepatocytes from liver tissue are best characterized in terms of nematic cell polarity [15]. We propose a visualization method for spatial patterns of nematic cell polarity in terms of equivalent cuboids. Applying this method to liver tissue reveals tissue-level patterns of coordinated cell polarity that follows a curved director field on the level of a liver lobule [15]. This spatial order may be difficult to detect by eye, prompting methods for accurate quantification.

To quantify orientational order in a three-dimensional tissue, we took inspiration from condensed matter physics. Specifically, we generalized the four biaxial orientational order parameters (OOP) *S*, *P*, *D*, *C* from the theory of liquid crystals [23, 28, 29], and generalized these as *co-orientational order parameters* (COOP). Traditionally, OOP are used to quantify the partial alignment of anisotropic molecules, where each molecules is characterized by a tripod of nematic axes [22, 30–32]. We propose COOP as a tool to quantify structural order in living matter. The COOP co−*S* characterizes the alignment of a nematic axis such as a cell polarity axis, relative to a given reference axis. If the fluctuations of the nematic axis around the reference axis are anisotropic, this results in non-zero values of the COOP co−*P*. The other two COOP co−*D* and co−*C* become relevant if the objects under consideration, e.g., polarized cells, are themselves biaxial, i.e., are characterized by a full tripod of nematic axes, instead of only a single axis. While a non-zero value of co−*D* indicates unexpected fluctuations of a second polarity axis (relative to the first reference axis), co−*C* characterizes preferential alignment of such a second polarity axis with a second reference axis. Here, we apply COOP to quantify the partial alignment of nematic cell polarity axes of hepatocytes in liver tissue.

The co-orientational order parameters (COOP) introduced here have several advantages: (i) unlike OOP, COOP do not depend on the choice of ordering of nematic axes and (ii) change continuously if system parameters are smoothly varied, yet are related to the classical OOP by simple linear transformations. Moreover, (iii) COOP can be applied to curved director fields, and (iv) can be generalized to the case of an ensemble of pairs of biaxial objects in a straightforward manner.

Applying these COOP to mouse liver tissue, we show that the liquid-crystal order of nematic cell polarity of hepatocytes is biaxial, which is a rare finding even in inanimate matter [33]. Furthermore, we found co-alignment between nematic cell polarity of hepatocytes and the local anisotropy of the sinusoidal network. This mutual alignment is not uniaxial, but of phase-biaxial type, i.e., its description requires two reference axes, a preferred axis of the sinusoidal network (approximately parallel to the direction of blood flow [26, 34]), and a plane axis, which characterizes local layered order of the sinusoidal network. Specifically, we observe signatures of layered organization of the sinusoidal network, characterized by a distinguished plane axis (**s**_2_), normal to layers enriched in sinusoids. Of note, this layered organization, with plane axis approximately parallel to the large veins, is different and goes beyond the ‘layered order’ previously reported in [15, Fig 3H], which we can now re-interpret as an additional organization *within* each sinusoidal layer (characterized, in our notation, by the third sinusoidal reference axis **s**_3_). Analyzing co-alignment between hepatocytes and the anisotropic sinusoidal network in terms of COOP revealed that the ring axis of apical cell polarity aligns on average parallel to the local plane axis of the sinusoidal network, while its fluctuations are biased away from the preferred axis. This biaxial order depends on communication between hepatocytes and sinusoidal cells. Re-analyzing data sets from [15], we find that COOP of hepatocyte polarity become substantially reduced in Integrin-*β*1 knockdown mice (see Fig S1E in S1G Appendix). This demonstrates the usefulness of COOP to quantitatively discriminate tissue phenotypes.

We conceptualized the observed biaxial order using a minimal interaction model, which quantitatively reproduces the COOP observed in the experimental data. The results from our minimal model provide insight into the previous observation of lobule-level spatial order of the bipolar axis (**a**_1_) of apical cell polarity of hepatocytes [15]. In fact, our minimal model suggests that it is primarily the ring axis (**a**_2_) that actively aligns relative to the local anisotropy of the sinusoidal network, from which the reported alignment of the bipolar axis (**a**_1_) follows as a corollary.

This biaxial liquid-crystal order may represent a self-organization principle of liver tissue that solves two conflicting design requirements [15]: every hepatocyte must be connected to both the sinusoidal network and the bile canaliculi network to fulfill its metabolic functions. One the other hand, the mutual distance between the two intertwined networks of sinusoids and bile canaliculi should be maximized [35], since oxygen- and nutrient-rich blood transported by sinusoids and toxic bile containing digestive enzymes transported by bile canaliculi should never mix. The layered organization of the sinusoidal network together with the nematic alignment of the ring axis (**a**_2_) of apical cell polarity parallel to the sinusoidal plane axis (**s**_2_) implies that ring-like surface patterns of apical cell membrane are approximately co-planar to the sinusoid layers: this maximizes the mutual distance between the sinusoidal and the bile canaliculi network (Fig 5B). Similarly, the anisotropic fluctuations of the ring axis away from the preferred sinusoid axis (**s**_1_) quantified by the phase-biaxiality COOP co−*P* > 0 implies that ring-like surface patterns are preferentially tilted in such a way that the “raised” and “lowered” points of a tilted ring pattern can “slip” between the sinusoids that run approximately in parallel in each sinusoid layer: this minimizes the risk that sinusoidal and bile canaliculi network get close. This structural order is not rigid and perfect, which should make it compatible with continuous turnover of the tissue by cell division and cell death. Remarkably, interfering with the communication between sinusoidal cells and hepatocytes disrupts this liquid-crystal order [15] with a concomitant reduction of COOP.

We anticipate that the co-orientational order parameters (COOP) introduced here can be useful to characterize local order also in a number of other biological systems, such as the cytoskeleton of individual cells, where local nematic order of cytoskeletal filaments couples to the generation of active stress [36] and thus cell shape and cell motility [2]. Next, our method could be applied to characterize collective cell migration, e.g., during embryonic development, wound healing and regeneration. Previously, nematic directors have been used to describe Planar Cell Polarity (PCP), as well as mechanical tension in mechanosensing processes in effectively two-dimensional tissues, which allowed identification of structure-function relationships [37]. To characterize fully three-dimensional tissues, new quantification tools like COOP will be needed. Moreover, quantification of local order by COOP can help to detect subtle alterations in disease states and genetic perturbations. Finally, with the advent of advanced three-dimensional imaging techniques for developing tissues such as light-sheet microscopy, COOP could be used to study organ development *in vivo* and in organoids.

## Supporting information

S1 Appendix Contains a list of mathematical symbols used (S1A), examples of raw image data (S1B), supplementary mathematical theory (S1C, S1I, S1J) supplementary analysis of spherical power spectra of kidney and hepatocyte cell polarity (S1D), details on numerical methods (S1E, S1F), supplementary analysis of biaxial order in murine liver from Integrin-*β*1 knock down mice (S1G), as well as two alternative nematic interaction models (S1H). (PDF)

S1 Matlab code Commented computer program that computes COOP for prototypical examples, including their visualization, as described in S1 Appendix S1K. (M)
