# Correction: Quantification of nematic cell polarity in three-dimensional tissues

**DOI:** 10.1371/journal.pcbi.1009349

**Published:** 2021-08-27

**Authors:** André Scholich, Simon Syga, Hernán Morales-Navarrete, Fabián Segovia-Miranda, Hidenori Nonaka, Kirstin Meyer, Walter de Back, Lutz Brusch, Yannis Kalaidzidis, Marino Zerial, Frank Jülicher, Benjamin M. Friedrich

The last author, Benjamin M. Friedrich, is incorrectly noted as the corresponding author. The correct corresponding author is Frank Jülicher. Dr. Jülicher’s email address is: julicher@pks.mpg.de.
