# Supplementary material for: Quantification of nematic cell polarity in three-dimensional tissues
Source: PLoS Comput Biol. 2020 Dec 10;16(12):e1008412. doi: 10.1371/journal.pcbi.1008412 (PMC7755288; doi:10.1371/journal.pcbi.1008412)
Supplement: S1 Appendix — (PDF) [file pcbi.1008412.s001.pdf]

S1 Appendix

Scholich et al.:

Quantification of Nematic Cell Polarity in Three-dimensional Tissues

S1A List of symbols.

Table S1A contains a list of symbols used in the main text.

S1B Typical examples of raw image data.

In supplementary Fig S1A, we show raw images of the example cells analyzed in Fig 1FG. In supplementary Fig S1B, we show raw images of the liver lobule shown in Fig 2DE and Fig 4AB.

S1C Spherical projection of membrane protein density.

We discuss two possible methods to project surface distributions $\rho(\mathbf{x})$ on an arbitrary star-convex surface onto a sphere, shown schematically in Fig S1C. The first method, depicted in panel A, retains the nominal value of the surface distribution. In the second method, shown in panel B, the local surface density $\rho(\mathbf{x})$ is weighted by the relative change in area upon projection. In this case, the total mass of the distribution is preserved. In the main text, we choose the first projection method because it ensures that a homogeneous distribution $\rho(\mathbf{x})$ on the cell surface yields a projected distribution $f(\mathbf{x})$ on the unit sphere that is again homogeneous. By that, the effect of cell shape on the projected distribution is greatly reduced. We confirmed that changing the projection method almost did not change computed cell polarity axes for most hepatocytes in liver tissue. We note that the first method (shown in Fig S1CA and used throughout this work), can be generalized to non star-convex cells, provided a distinguished cell center can still be defined for each cell, by using a maximum projection. In some potential applications, e.g., the study of planar cell polarity (PCP), actual intensities of polarity markers are important, which provides an additional argument in favor of the first method.

We used the projection method described above to compute spherical power spectra of apical membrane distributions for kidney cells and hepatocytes, see Fig 1FG in the

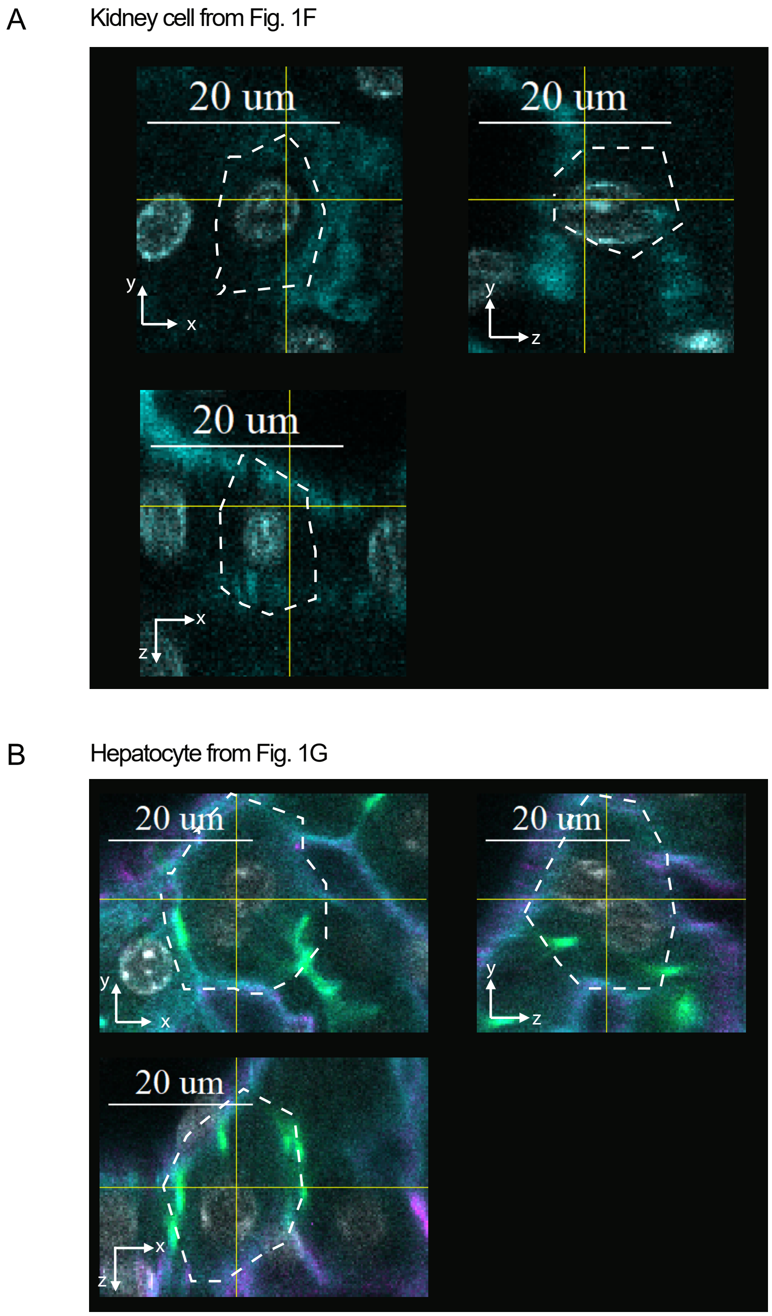

Fig S1A. Example raw images of cells analyzed in Figure 1F and 1G. (A) Close-up of high-resolution imaging of an adult mouse kidney section stained for nucleus (DAPI, gray) and cell borders (phalloidin, cyan), represented as orthogonal views. The outline of the kidney cell analyzed in Fig 1F is indicated by a dashed white line. (B) Same as panel A, but for an adult mouse liver section stained for bile canaliculi (CD13, green), sinusoids (fibronectin and laminin, magenta), nucleus (DAPI, gray) and cell border (phalloidin, cyan). The example hepatocyte analyzed in Fig 1G is indicated by a dashed white line. Tissue sections were optically cleared and imaged at high resolution using multiphoton microscopy ($0.3\,\mu m \times 0.3\,\mu m \times 0.3\,\mu m$ per voxel). Methods as described in [15].

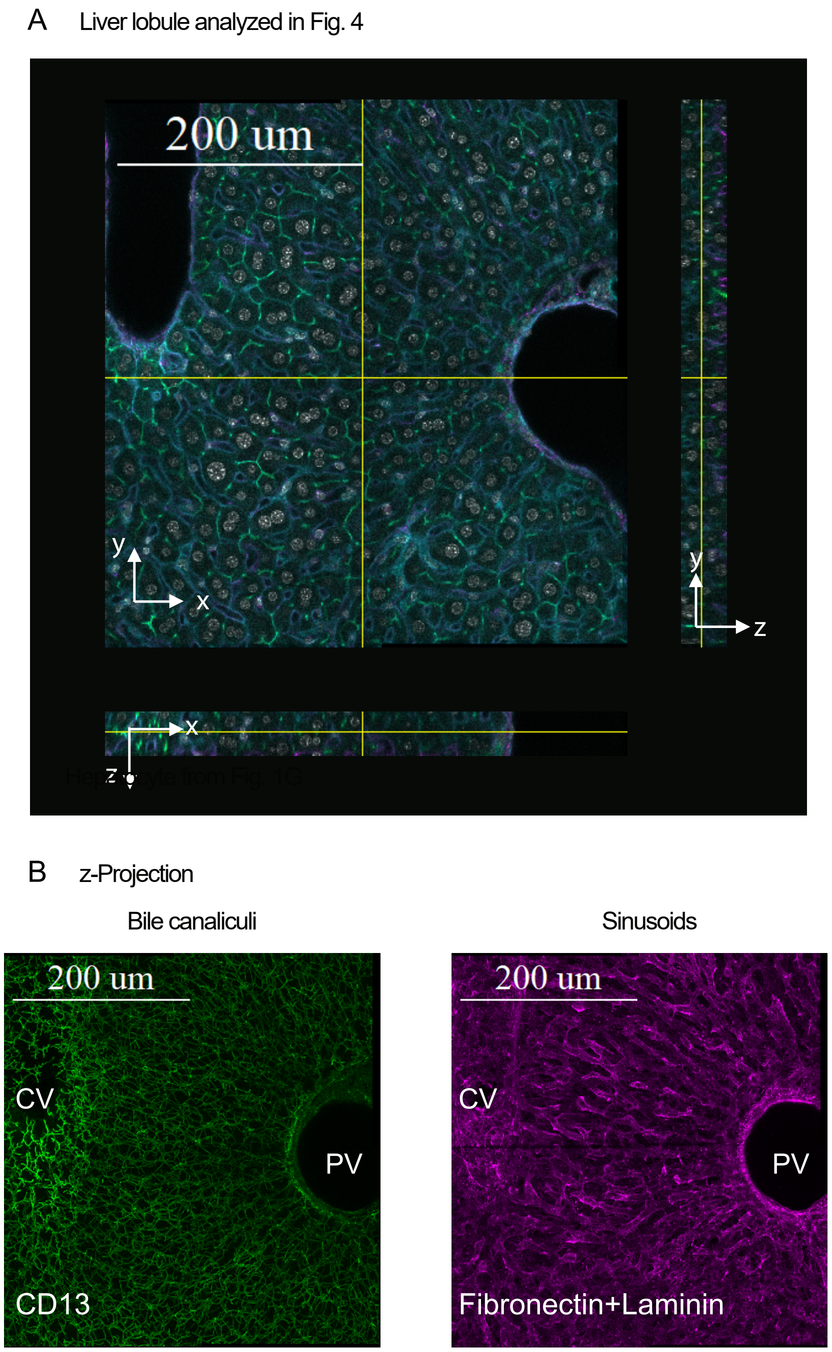

Fig S1B. Raw image of the liver lobule shown in Figure 4A and 4B. (A) High-resolution imaging of an adult mouse liver section (thickness $\approx 80\,\mu$m) stained for bile canaliculi (CD13, green), sinusoids (fibronectin and laminin, magenta), nucleus (DAPI, gray) and cell border (phalloidin, cyan), represented as orthogonal views. (B) Maximal intensity z-projection of the same tissue slice stained for bile canaliculi (left) and sinusoids (right). Methods as described in [15].

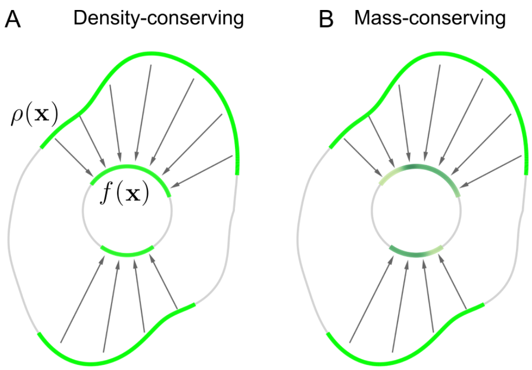

Fig S1C. Schematic of spherical projection methods.
We illustrate two methods to radially project a surface density (indicated in green) on a star-convex domain onto a co-centric sphere. (A) In the variant used in the main text, the nominal value of the surface density is retained. (B) Alternatively, one could multiply the local surface density by the relative change in area upon projection. Thus, the total mass of the distribution is conserved. However, the resultant spherical distribution will confound anisotropy of the original distribution and anisotropy of domain shape.

main text. In supplementary Fig S1D, we provide additional statistical information for the respective spherical power spectra.

S1D Relation between second mode spherical spectral power and order parameters.

We can consider a distribution $p(\mathbf{n})$ of nematic axes $\mathbf{n}$, represented by antipodal pairs of points, also as a surface distribution on the unit sphere, which is symmetric with respect to a point reflection at the center. Under this correspondence, the uniaxial order parameters S and P for $\mathbf{n}$ as first principal axis are intimately linked to the expansion of $p(\mathbf{n})$ into spherical harmonics, Eq. (1).

Specifically, we interpret $p(\mathbf{n})$ as the density $f(\mathbf{x}) = p(\mathbf{n}) + p(-\mathbf{n})$ defined on the unit sphere $\mathcal{S}^2$. For sake of reference, we recall the decomposition of $f(\mathbf{x})$ into spherical harmonics

$$f(\mathbf{x}) = \sum_{l=0}^{\infty} F(\mathbf{x}) = \sum_{l=0}^{\infty} \sum_{m=-l}^{l} f_l^m Y_l^m(\mathbf{x}) \quad , \tag{S1}$$

where $F_l(\mathbf{x}) = \sum_{m=-l}^{l} f_l^m Y_l^m(\mathbf{x})$ and the normalized spherical harmonics $Y_l^m(\mathbf{x})$ are given in terms of spherical coordinates with polar angle θ and azimuthal angle φ

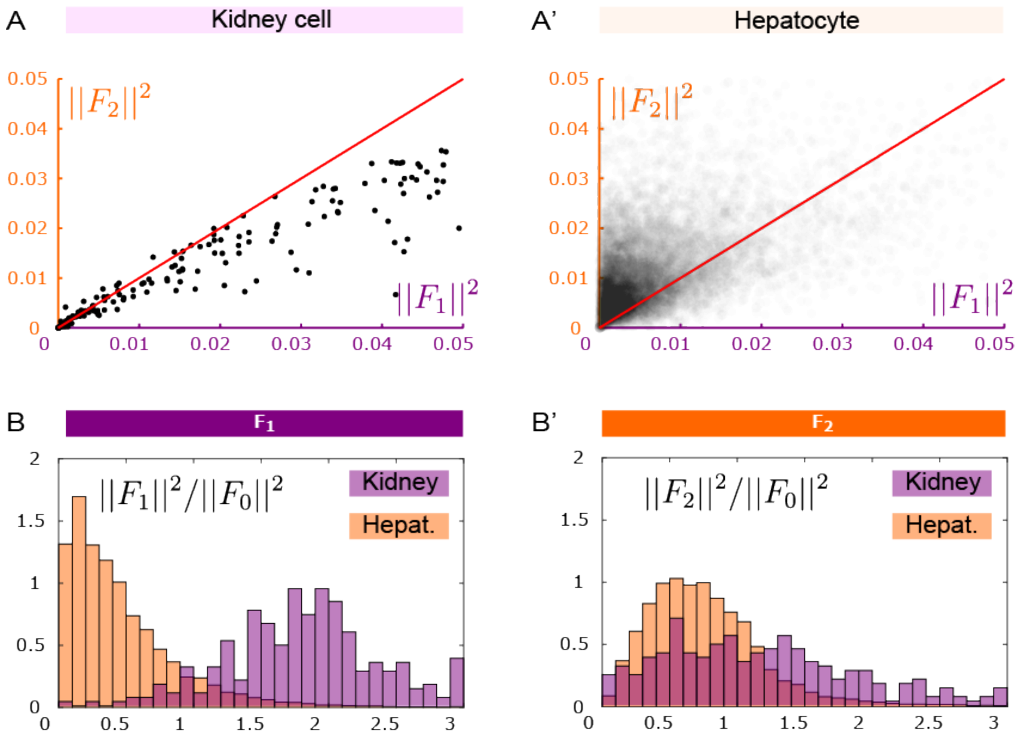

Fig S1D. Quantification of spherical power spectra for kidney cells and hepatocytes.

(A) Scatter plot of first and second spherical Fourier coefficient for apical membrane distribution of kidney cells ($n = 286$ cells). For 79% of kidney cells, $||F_1||^2 > ||F_2||^2$, corresponding to points *below* the red diagonal. (A') Same as panel A but for hepatocytes ($n = 9983$ cells; data points are shown transparent due to their large number). For 75% of hepatocytes, $||F_1||^2 < ||F_2||^2$, corresponding to points *above* the red diagonal. (B) Histogram of normalized first spherical Fourier coefficients $||F_1||^2/||F_0||^2||$ for kidney cells (magenta) and hepatocytes (orange), revealing a characteristic unimodular distribution of $||F_1||^2/||F_0||^2||$ for kidney cells. (B') Same as panel B for the second spherical Fourier coefficients $||F_2||^2/||F_0||^2||$, revealing a characteristic unimodular distribution of $||F_2||^2/||F_0||^2||$ for hepatocytes. (Histogram normalized as probability density function.)

(relative to the chosen z axis) by

$$Y_l^m = \sqrt{\frac{(2l+1)(l-m)!}{4\,\pi\;(l+m)!}}\,P_l^m(\cos\theta)\,e^{i\,m\varphi} \quad . \tag{S2}$$

Here, P_l^m denote the associated Legendre polynomials.

We assume that $p(\mathbf{n})$ possesses D_{2h}-symmetry. Note that all odd spherical modes $F_{2l+1}(\mathbf{x})$ vanish in this case. The second spherical mode $F_2(\mathbf{x})$ is a nematic tensor whose mutually perpendicular eigenvectors define reference axes $\mathbf{u}$, $\mathbf{v}$, $\mathbf{w}$, see main text on page 12. Without loss of generality, the reference axes $\mathbf{u}$, $\mathbf{v}$, $\mathbf{w}$ shall be aligned with the x, y, z axis, respectively. Then, all coefficients f_2^m of the second mode $F_2(\mathbf{x})$ with odd index m must vanish i.e., $f_2^{-1} = f_2^1 = 0$ and $\mathrm{Im} f_2^{-2} = \mathrm{Im} f_2^2 = 0$, because these correspond to functions that are odd relative to a line reflection at the z-axis. Moreover, $f_2^2 = f_2^{-2}$, since $p(\mathbf{n})$ is real.

We thus find [21]

$$S = \sqrt{\frac{4\,\pi}{5}} f_2^0 \qquad P = \sqrt{6\,\frac{4\,\pi}{5}} f_2^2 \tag{S3}$$

Conversely, the spherical power in the second mode can be expressed in terms of S and P

$$S_{ff}(2) = ||F_2||^2 = \frac{1}{4\pi}\sum_{m=-2}^{2} |f_2^m|^2 = \frac{5}{(4\pi)^2}\left(S^2 + \frac{1}{3}P^2\right) \quad . \tag{S4}$$

S1E Cuboid visualization of nematic cell polarity.

In the main text, we present a method to visualize nematic tensors $\boldsymbol{A}$ by colored cuboids as shown in Fig 2. We provide additional details on this method. For a surface density $f(\mathbf{x})$ on the unit sphere $\mathcal{S}^2$, the moments-of-inertia tensor $\boldsymbol{I}$ reads

$$\boldsymbol{I} = \int_{\mathcal{S}^2} \mathrm{d}^2\mathbf{x}\;(\mathbb{1} - \mathbf{x}\otimes\mathbf{x})\;\rho(\mathbf{x}), \tag{S5}$$

i.e.,

$$\boldsymbol{I} = \frac{2}{3}\,(F_0\mathbb{1} - \boldsymbol{A}) \quad , \tag{S6}$$

where $\boldsymbol{A}$ is the nematic tensor associated to $f(\mathbf{x})$, see Eq. (3), and $F_0 = \int_{\mathcal{S}^2} \mathrm{d}^2\mathbf{x}\,\rho(\mathbf{x})$. Both tensors diagonalize in the same eigenframe. The eigenvalues ι_1, ι_2, ι_3 of $\boldsymbol{I}$ (called principal moments of inertia), and the eigenvalues α_1, α_2, α_3 of $\boldsymbol{A}$ are related by $\iota_i = (2/3)F_0 - \alpha_i$, $i = 1, 2, 3$ (for a suitable ordering of ι_i).

In turn, the principal moments of inertia ι_i of a solid cuboid with side lengths a, b, c are given by $\iota_1 = (b^2 + c^2)/12$, $\iota_2 = (a^2 + b^2)/12$, $\iota_3 = (a^2 + c^2)/12$. Using Eq. (S6), we find the side-lengths a, b, c of a cuboid that has the same principal moments of inertia as $\boldsymbol{I}$

$$a^2 = 6\left(\frac{2}{3}F_0 + \alpha_1 - \alpha_2 - \alpha_3\right) \quad , \tag{S7}$$

$$b^2 = 6\left(\frac{2}{3}F_0 + \alpha_3 - \alpha_1 - \alpha_2\right) \quad , \tag{S8}$$

$$c^2 = 6\left(\frac{2}{3}F_0 + \alpha_2 - \alpha_3 - \alpha_1\right) \quad . \tag{S9}$$

In plots, cuboids are rescaled by a constant factor.

S1F Gaussian average of nematic tensors.

The coarse-grained orientation patterns shown in Fig 2E are calculated from the nematic tensors $\boldsymbol{A}$ of individual hepatocytes by averaging with a Gaussian kernel. Specifically, given nematic tensors $\boldsymbol{A}^{(i)}$ at cell center locations $\mathbf{x}^{(i)}$, the coarse-grained tensor $\langle \boldsymbol{A} \rangle_{\mathrm{loc}}(\mathbf{x})$ at location $\mathbf{x}$ is calculated by

$$\langle \boldsymbol{A} \rangle_{\mathrm{loc}}(\mathbf{x}) = \sum_{i \neq j} \frac{1}{(2\pi\,\sigma^2)^{3/2}} \exp\left(-\frac{|\mathbf{x}^{(i)} - \mathbf{x}|^2}{2\,\sigma^2}\right) \boldsymbol{A}^{(i)} \quad . \tag{S10}$$

Here, σ denotes the standard deviation of the Gaussian kernel, which sets the length-scale of coarse-graining. Note that we used a “punctured” Gaussian averaging kernel that omits the tensor of the central cell, thereby avoiding any bias. As a side-node, instead of averaging nematic tensors $\boldsymbol{A}$, each derived from an individual surface distribution $f^{(i)}(\mathbf{x})$, we could have equivalently averaged the surface distributions first, and then computed $\langle \boldsymbol{A} \rangle_{\mathrm{loc}}$ as the nematic tensor of an averaged surface distribution $\langle f(\mathbf{x}) \rangle_{\mathrm{loc}}$. For the principal axes of the averaged tensor $\langle \boldsymbol{A} \rangle_{\mathrm{loc}}$, we write $\langle \mathbf{a}_1 \rangle_{\mathrm{loc}}$, $\langle \mathbf{a}_2 \rangle_{\mathrm{loc}}$, $\langle \mathbf{a}_3 \rangle_{\mathrm{loc}}$, for short.

S1G Co-orientational order parameters for Integrin-β1.

In a previous publication, we had shown that selective silencing of Integrin-β1 in hepatocytes of murine liver substantially reduces liquid-crystal order of hepatocyte polarity [15]. We have re-analyzed the same data in terms of the new co-orientational order parameters (COOP) introduced in this work. We find that in Integrin-β1 knockdown mice, COOP are substantially reduced both for COOP that compare hepatocyte polarity with reference axes provided by the local average of hepatocyte polarity itself, and for COOP that compare hepatocyte polarity to the local anisotropy of the sinusoidal network, see Fig S1EDF. In contrast, control conditions (siRNA against Luciferase) gave as essentially the same COOP as adult data sets without siRNA. Thus, silencing Integrin-β1, which affects communication between hepatocytes and sinusoidal cells, perturbs the tissue-level alignment of hepatocyte polarity as well as co-alignment with the sinusoidal network.

S1H Nematic interaction models.

For efficient simulation of the nematic interaction model given by Eq. (12), we used a constant nematic tensor for the anisotropy of the sinusoidal network, $\boldsymbol{S} = \sigma_1\,\mathbf{s}_1 \otimes \mathbf{s}_1 + \sigma_2\,\mathbf{s}_2 \otimes \mathbf{s}_2 + \sigma_3\,\mathbf{s}_3 \otimes \mathbf{s}_3$, with eigenvectors $\mathbf{s}_1$, $\mathbf{s}_2$, $\mathbf{s}_3$, and corresponding eigenvalues σ_1, σ_2, σ_3. The eigenvalues σ_1, σ_2, σ_3 of $\boldsymbol{S}$ were computed as the average of the respective eigenvalues of the local anisotropy tensors $\boldsymbol{S}(\mathbf{x}^{(i)})$ given by Eq. (11). For the mouse liver tissue data set analyzed here, these eigenvalues read $\sigma_1 \approx 0.22$, $\sigma_2 \approx -0.19$, $\sigma_3 = -\sigma_1 - \sigma_2$. Likewise, we computed average eigenvalues of the tensor $\boldsymbol{A}$ of apical cell polarity of hepatocytes as $\alpha_1 \approx 0.18$, $\alpha_2 \approx -0.19$, $\alpha_3 = -\alpha_1 - \alpha_2$.

In addition to the interaction proposed in Eq. (12), two model variants are conceivable: (a) the bipolar axis $\mathbf{a}_1$ of hepatocytes could be coupled to the local anisotropy tensor $\boldsymbol{S}$ of the sinusoidal network, or (b) the full nematic tensor $\boldsymbol{A}$ of hepatocyte polarity, which comprises both the ring and the bipolar axes, could couple to $\boldsymbol{S}$. These two model variants (a) and (b) correspond to the respective effective interaction energies

$$\text{model variant (a):} \quad H = -\lambda\,\alpha_1\,(\mathbf{a}_1 \otimes \mathbf{a}_1) : \boldsymbol{S} \tag{S11a}$$

$$\text{model variant (b):} \quad H = -\lambda\,\boldsymbol{A} : \boldsymbol{S} \tag{S11b}$$

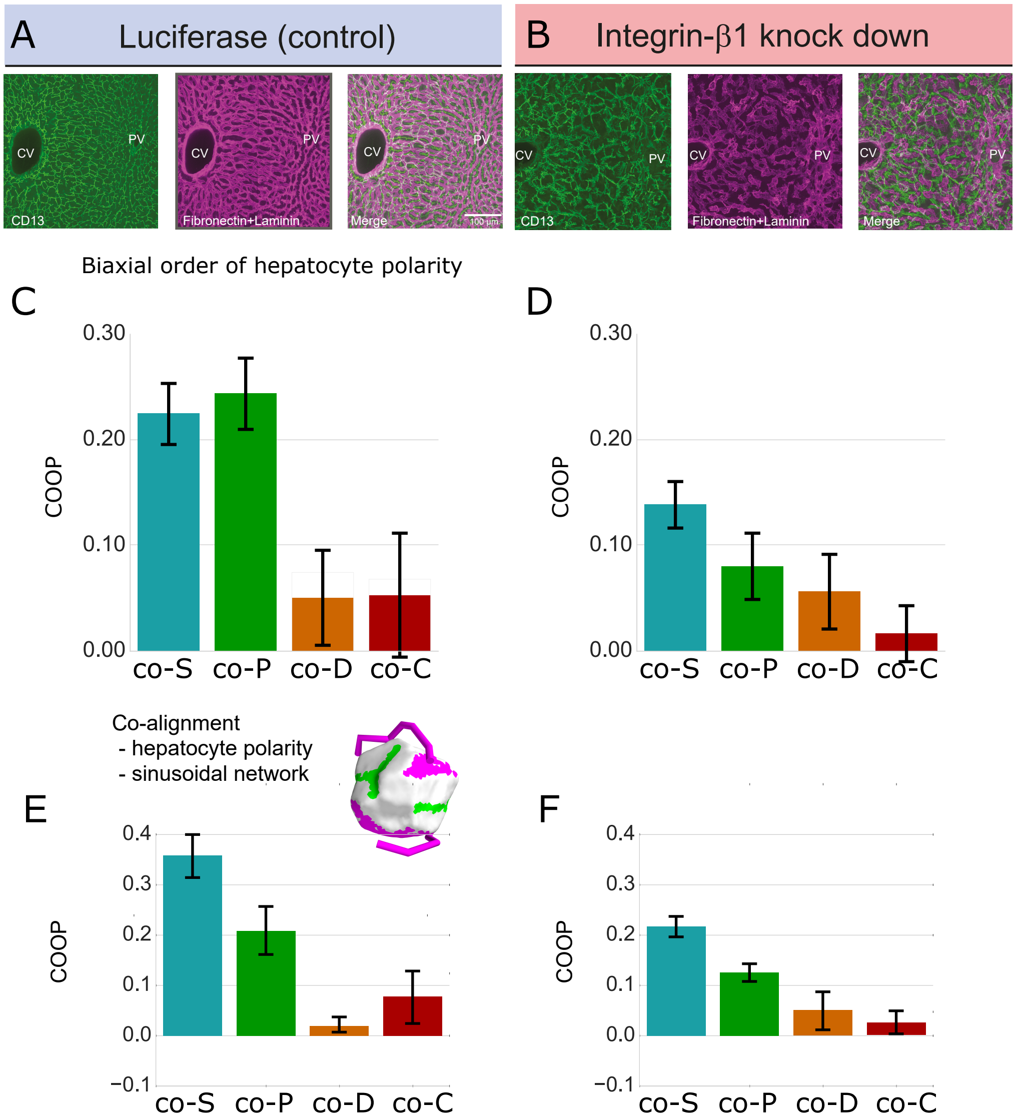

Fig S1E. COOP of hepatocyte polarity are reduced in Integrin-β1 KD mice. (A) Maximal intensity z-projection of a liver tissue slice from control mice (siRNA against Luciferase) stained for bile canaliculi (left), sinusoids (middle), and merge (right). Tissue organization is indistinguishable from normal adult tissue. Methods as described in [15]. (B) Same as panel A, but for Integrin-β1 knockdown mice. The bile canaliculi network and the sinusoidal network each display reduced apparent alignment with the CV-PV axis in comparison to control conditions. Panels A and B reproduced from [15] under CC-BY license. (C) Co-orientational order parameters (COOP) quantifying hepatocyte polarity with respect to reference axes provided by the local average of hepatocyte polarity itself, analogous to Fig 3G in the main text, but for control conditions (siRNA against Luciferase, mean±s.d., $n = 7$ tissue samples). (D) Same as panel C, but for Integrin-β1 knockdown mice, revealing a substantial reduction of co-S and co-P in comparison to control conditions (mean±s.d., $n = 4$ tissue samples). (E) Co-orientational order parameters (COOP) quantifying hepatocyte polarity with respect to reference axes provided by the local anisotropy of the sinusoidal network analogous to Fig 4C in the main text, but for control conditions. (F) Same as panel E, but for Integrin-β1 knockdown mice, revealing again a substantial reduction of co-S and co-P in comparison to control conditions (mean±s.d., $n = 4$ tissue samples).

Fig S1F shows simulation results for these two alternative models. We find that these alternative models cannot account for the experimentally observed values of the co-orientational order parameters.

S1I Effect of axes permutations on orientational order parameters S, P, D, C

The orientational order parameters (OOP) S, P, D, C defined in Eq. (7) change under a permutation $\pi \in S_3$ of the principal axes, $\mathbf{n} = \mathbf{a}_{\pi(2)}$, $\mathbf{m} = \mathbf{a}_{\pi(1)}$, $\mathbf{l} = a_{\pi(3)}$, as well as under a permutation $\rho \in S_3$ of the reference axes, $\mathbf{w} = \mathbf{e}_{\rho(2)}$, $\mathbf{v} = \mathbf{e}_{\rho(1)}$, $\mathbf{u} = \mathbf{e}_{\rho(3)}$. The action of the direct product of both permutation groups, $G = S_3 \times S_3$, defines an equivalence relation on the four-dimensional space of 4-tuples (S, P, D, C), where each G-orbit defines one equivalence class that corresponds to the same state of orientational order. Fig S1G illustrates the action of the permutation group G on a two-dimensional section of the four-dimensional (S, P, D, C)-space.

Table S1B lists the transformation of the orientational order parameters (OOP) S, P, D, C under the action of the group G.

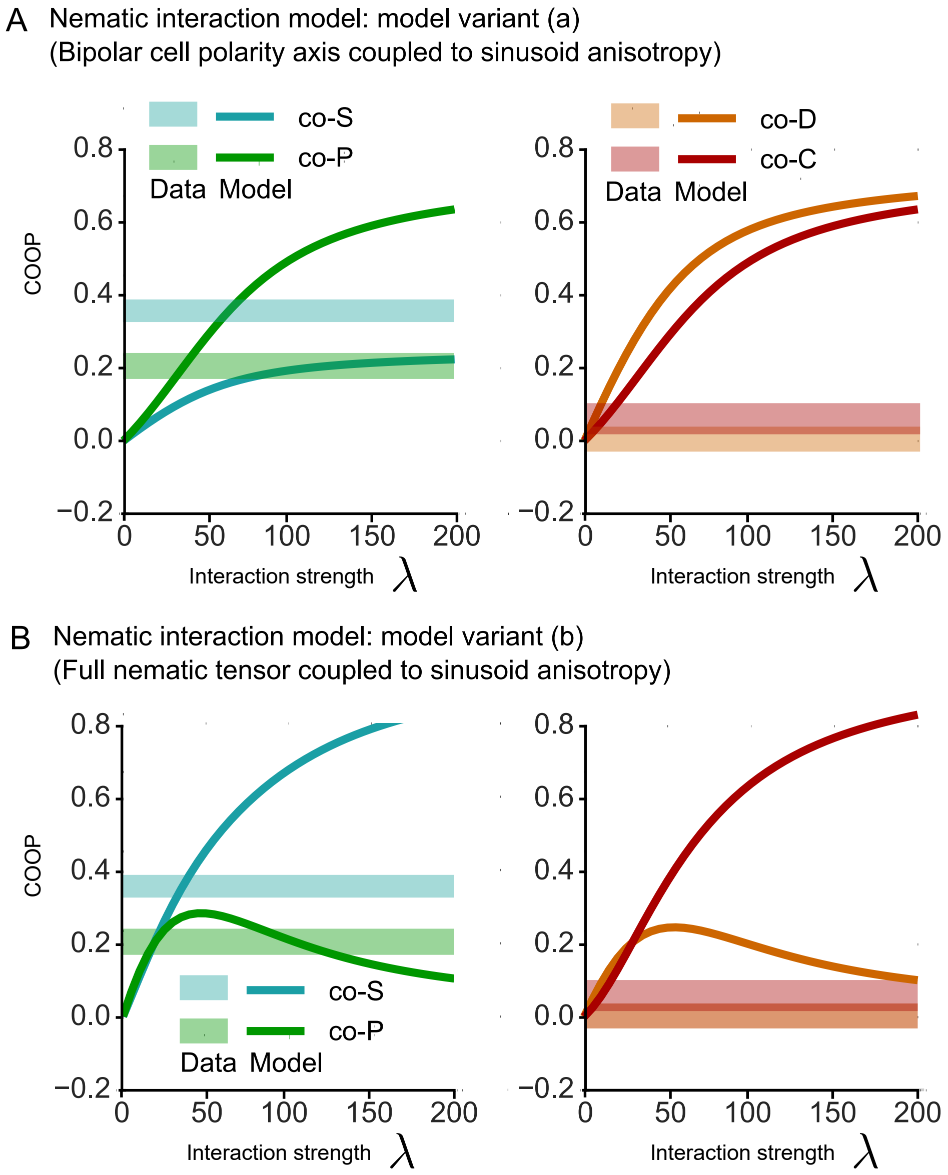

Fig S1F. Alternative minimal interaction models ruled out by experimental data. (A) Co-orientational order parameters predicted by a variant of the minimal interaction model, where only the bipolar apical nematic axis $\mathbf{a}_1$ of hepatocytes is coupled to the anisotropy of the local sinusoidal network, see Eq. (S11a). There exists no value of the effective interaction strength λ for which simulation results are consistent with the experimental values (shaded region: mean±s.d., $n = 12$ tissue samples). (B) Co-orientational order parameters predicted by a second variant of the minimal interaction model, see Eq. (S11b). Here, the full apical nematic polarity tensor $\boldsymbol{A}$ of hepatocytes is coupled to the anisotropy of the local sinusoidal network. Again, there exists no value of λ consistent with the experimental data.

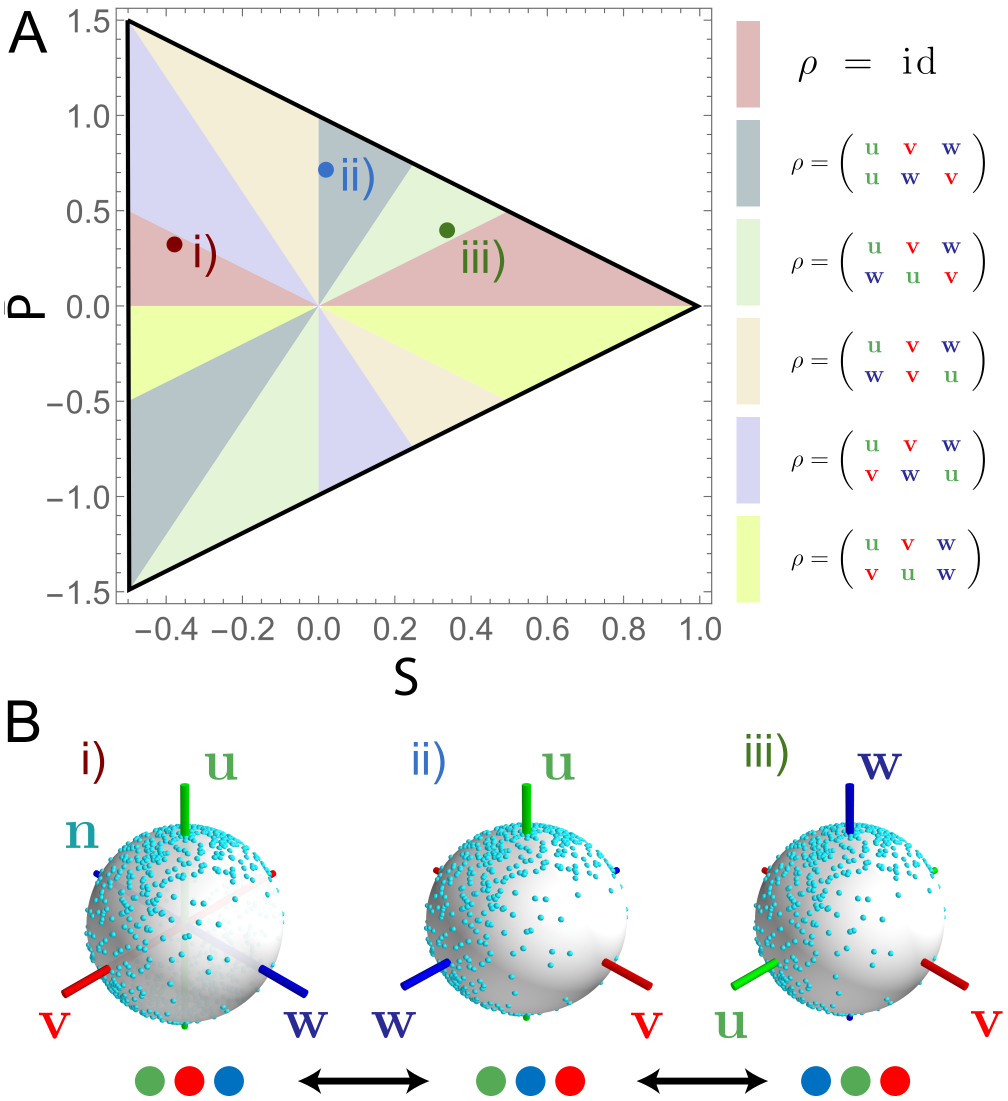

Fig S1G. Action of axes permutation on orientational order parameters (OOP). (A) Illustration of the action of the permutation group $G = S_3 \times S_3$ (which permutes principal and reference axes, each) on the four-dimensional (S, P, D, C)-space of orientational order parameters. Shown is the section defined by $D = C = 0$, corresponding to the subspace spanned by S and P that describes orientational order of a single axis. Colored regions show the tessellation of this subspace under the action of the permutation group S_3 of reference axes $\mathbf{u}$, $\mathbf{v}$, $\mathbf{w}$. The red region containing case i) corresponds to a common convention in the theory of liquid crystals [21, 23]. (B) Example distribution of first principal axis $\mathbf{n}$ (represented by antipodal pairs of cyan points on the unit sphere), displaying phase-biaxial order. Three different permutations of the reference axes (cases i, ii, iii) give rise to three different sets of order parameters for the same distribution of principal axes (corresponding values of co-S and co-P indicated in panel A).

Table S1A. List of symbols used in the main text.

Symbol	Description
$\rho(\mathbf{x})$	scalar area density, e.g., density of polarity marker on cell surface
$f(\mathbf{x})$	scalar area density on unit sphere $\mathcal{S}^2$; projection of $\rho(\mathbf{x})$
$F_l(\mathbf{x})$	l-th mode of spherical Fourier transform of $f(\mathbf{x})$, see Eq. (1)
f_l^m	m-th expansion coefficient of $F_l(\mathbf{x})$, for expansion into spherical harmonics
$\mathcal{S}^2$	unit sphere
$\int_{\mathcal{S}^2} \mathrm{d}^2\mathbf{x}$	integral over unit sphere, using standard Euclidean measure
$\lvert\lvert F_l \rvert\rvert^2$	spherical power of l-th mode $F_l(\mathbf{x})$ of spherical Fourier transform of $f(\mathbf{x})$, see Eq. (2)
$\boldsymbol{A}$	nematic tensor associated to surface density $f(\mathbf{x})$, see Eq. (3)
α_1, α_2, α_3	eigenvalues of $\boldsymbol{A}$
$\mathbf{a}_1$, $\mathbf{a}_2$, $\mathbf{a}_3$	eigenvectors of $\boldsymbol{A}$ corresponding to α_1, α_2, α_3 with $\alpha_1 \geq \alpha_3 \geq \alpha_2$; we refer to $\mathbf{a}_1$ as *bipolar axis* and $\mathbf{a}_2$ as *ring axis*
$\mathbf{n}$, $\mathbf{m}$, $\mathbf{l}$	principal axes; the tripod of ortho-normal vectors $\mathbf{n}$, $\mathbf{m}$, $\mathbf{l}$ represents a permutation of $\mathbf{a}_1$, $\mathbf{a}_2$, $\mathbf{a}_3$: $\mathbf{n} = \mathbf{a}_{\pi(2)}$, $\mathbf{m} = \mathbf{a}_{\pi(3)}$, $\mathbf{n} = \mathbf{a}_{\pi(1)}$ with $\mathbf{n}$ first principal axis, $\mathbf{m}$ second principal axis, $\mathbf{l}$ third principal axis
$\pi, \rho \in S_3$	permutations of the indices $(1, 2, 3)$
$\boldsymbol{Q}$, $\boldsymbol{B}$	traceless tensors, which characterize the tripod $\mathbf{n}$, $\mathbf{m}$, $\mathbf{l}$, see Eq. (4)
$\boldsymbol{R}_Q$, $\boldsymbol{R}_B$	rotation matrices that diagonalize $\boldsymbol{Q}$ and $\boldsymbol{B}$, respectively, see Eqs. (5,6)
$\mathbf{w}$, $\mathbf{v}$, $\mathbf{u}$	reference axes; derived from either a common eigenframe $\mathbf{e}_1$, $\mathbf{e}_2$, $\mathbf{e}_3$ of the tensors $\boldsymbol{Q}$ and $\boldsymbol{B}$, $\mathbf{w} = \mathbf{e}_{\rho(2)}$, $\mathbf{v} = \mathbf{e}_{\rho(3)}$, $\mathbf{u} = \mathbf{e}_{\rho(1)}$ (OOP), or from a second set of nematic tensors $\boldsymbol{E}$ with eigenvalues ε_1, ε_2, ε_3 that are ordered such that $\varepsilon_1 \geq \varepsilon_3 \geq \varepsilon_2$, and corresponding eigenvectors $\mathbf{e}_1$, $\mathbf{e}_2$, $\mathbf{e}_3$: $\mathbf{w} = \mathbf{e}_2$, $\mathbf{v} = \mathbf{e}_3$, $\mathbf{u} = \mathbf{e}_1$ (COOP); in both cases, we refer to the orthonormal vectors $\mathbf{w}$, $\mathbf{v}$, $\mathbf{u}$ as $\mathbf{w}$ first reference axis, $\mathbf{v}$ second reference axis, $\mathbf{u}$ third reference axis
S, P, D, C	orientational order parameters (OOP) that characterize biaxial order in an ensemble of tripods $\mathbf{a}_1^{(i)}$, $\mathbf{a}_2^{(i)}$, $\mathbf{a}_3^{(i)}$ (with D_{2h}-symmetry), indexed by i; S is the (uniaxial) nematic order parameter, P is the phase-biaxial order parameter, D and C are molecular biaxiality parameters that quantify order of a second nematic axis, see Eq. (7)
co-S, co-P, co-D, co-C	co-orientational order parameters (COOP), introduced here, corresponding to a fixed ordering of principal axes, $\mathbf{n} = \mathbf{a}_2$, $\mathbf{m} = \mathbf{a}_3$, $\mathbf{l} = \mathbf{a}_1$, derived from the eigenvectors $\mathbf{a}_1$, $\mathbf{a}_2$, $\mathbf{a}_3$ of a nematic tensor $\boldsymbol{A}$ with corresponding eigenvalues α_1, α_2, α_3 that are ordered such that $\alpha_1 \geq \alpha_3 \geq \alpha_2$, and a fixed ordering of reference axes $\mathbf{w} = \mathbf{e}_2$, $\mathbf{v} = \mathbf{e}_3$, $\mathbf{u} = \mathbf{e}_1$ derived from the eigenvectors $\mathbf{e}_1$, $\mathbf{e}_3$, $\mathbf{e}_2$ of a second nematic tensor $\boldsymbol{E}$ with corresponding eigenvalues ε_1, ε_2, ε_3 that are ordered such that $\varepsilon_1 \geq \varepsilon_3 \geq \varepsilon_2$, see Eq. (10)
$\boldsymbol{S}$	nematic tensor of local anisotropy of sinusoidal network, see Eq. (11)
$\mathbf{s}_1$, $\mathbf{s}_2$, $\mathbf{s}_3$	eigenvectors of $\boldsymbol{S}$, corresponding to eigenvalues ε_1, ε_2, ε_3 with $\varepsilon_1 \geq \varepsilon_3 \geq \varepsilon_2$
$\mathbb{1}$	identity tensor with components $\mathbb{1}_{\alpha\beta} = \delta_{\alpha\beta}$
H	dimensionless Hamiltonian of minimal interaction model, see Eq. (12)
λ	effective interaction strength in H
$\boldsymbol{I}$	moments-of-inertia tensor, see Eq. (S5)
a, b, c	side-lengths of equivalent cuboid for the visualization of nematic tensors $\boldsymbol{A}$, see SI text S1E; the convention $\alpha_1 \geq \alpha_3 \geq \alpha_2$ for the eigenvalues α_1, α_2, α_3 of $\boldsymbol{A}$ implies $a \geq b \geq c$; faces normal to edges of length a are colored red, faces normal to edges of length b are colored green, faces normal to edges of length c are colored blue

π	ρ	S	P	D	C
**(lmn)**	**(uvw)**	S	P	D	C
	**(uwv)**	$\frac{1}{2}(-S-P)$	$\frac{1}{2}(-3S+P)$	$\frac{1}{2}(-D-3C)$	$\frac{1}{2}(-D+C)$
	**(vuw)**	S	$-P$	D	$-C$
	**(vwu)**	$\frac{1}{2}(-S-P)$	$\frac{1}{2}(3S-P)$	$\frac{1}{2}(-D-3C)$	$\frac{1}{2}(D-C)$
	**(wuv)**	$\frac{1}{2}(-S+P)$	$\frac{1}{2}(-3S-P)$	$\frac{1}{2}(-D+3C)$	$\frac{1}{2}(-D-C)$
	**(wvu)**	$\frac{1}{2}(-S+P)$	$\frac{1}{2}(3S+P)$	$\frac{1}{2}(-D+3C)$	$\frac{1}{2}(D+C)$
**(lnm)**	**(uvw)**	$\frac{1}{2}(-S-D)$	$\frac{1}{2}(-P-3C)$	$\frac{1}{2}(-3S+D)$	$\frac{1}{2}(-P+C)$
	**(uwv)**	$\frac{1}{4}(S+P+D+3C)$	$\frac{1}{4}(3S-P+3D-3C)$	$\frac{1}{4}(3S+3P-D-3C)$	$\frac{1}{4}(3S-P-D+C)$
	**(vuw)**	$\frac{1}{2}(-S-D)$	$\frac{1}{2}(P+3C)$	$\frac{1}{2}(-3S+D)$	$\frac{1}{2}(P-C)$
	**(vwu)**	$\frac{1}{4}(S+P+D+3C)$	$\frac{1}{4}(-3S+P-3D+3C)$	$\frac{1}{4}(3S+3P-D-3C)$	$\frac{1}{4}(-3S+P+D-C)$
	**(wuv)**	$\frac{1}{4}(S-P+D-3C)$	$\frac{1}{4}(3S+P+3D+3C)$	$\frac{1}{4}(3S-3P-D+3C)$	$\frac{1}{4}(3S+P-D-C)$
	**(wvu)**	$\frac{1}{4}(S-P+D-3C)$	$\frac{1}{4}(-3S-P-3D-3C)$	$\frac{1}{4}(3S-3P-D+3C)$	$\frac{1}{4}(-3S-P+D+C)$
**(mln)**	**(uvw)**	S	P	$-D$	$-C$
	**(uwv)**	$\frac{1}{2}(-S-P)$	$\frac{1}{2}(-3S+P)$	$\frac{1}{2}(D+3C)$	$\frac{1}{2}(D-C)$
	**(vuw)**	S	$-P$	$-D$	C
	**(vwu)**	$\frac{1}{2}(-S-P)$	$\frac{1}{2}(3S-P)$	$\frac{1}{2}(D+3C)$	$\frac{1}{2}(-D+C)$
	**(wuv)**	$\frac{1}{2}(-S+P)$	$\frac{1}{2}(-3S-P)$	$\frac{1}{2}(D-3C)$	$\frac{1}{2}(D+C)$
	**(wvu)**	$\frac{1}{2}(-S+P)$	$\frac{1}{2}(3S+P)$	$\frac{1}{2}(D-3C)$	$\frac{1}{2}(-D-C)$
**(mnl)**	**(uvw)**	$\frac{1}{2}(-S-D)$	$\frac{1}{2}(-P-3C)$	$\frac{1}{2}(3S-D)$	$\frac{1}{2}(P-C)$
	**(uwv)**	$\frac{1}{4}(S+P+D+3C)$	$\frac{1}{4}(3S-P+3D-3C)$	$\frac{1}{4}(-3S-3P+D+3C)$	$\frac{1}{4}(-3S+P+D-C)$
	**(vuw)**	$\frac{1}{2}(-S-D)$	$\frac{1}{2}(P+3C)$	$\frac{1}{2}(3S-D)$	$\frac{1}{2}(-P+C)$
	**(vwu)**	$\frac{1}{4}(S+P+D+3C)$	$\frac{1}{4}(-3S+P-3D+3C)$	$\frac{1}{4}(-3S-3P+D+3C)$	$\frac{1}{4}(3S-P-D+C)$
	**(wuv)**	$\frac{1}{4}(S-P+D-3C)$	$\frac{1}{4}(3S+P+3D+3C)$	$\frac{1}{4}(-3S+3P+D-3C)$	$\frac{1}{4}(-3S-P+D+C)$
	**(wvu)**	$\frac{1}{4}(S-P+D-3C)$	$\frac{1}{4}(-3S-P-3D-3C)$	$\frac{1}{4}(-3S+3P+D-3C)$	$\frac{1}{4}(3S+P-D-C)$
**(nlm)**	**(uvw)**	$\frac{1}{2}(-S+D)$	$\frac{1}{2}(-P+3C)$	$\frac{1}{2}(-3S-D)$	$\frac{1}{2}(-P-C)$
	**(uwv)**	$\frac{1}{4}(S+P-D-3C)$	$\frac{1}{4}(3S-P-3D+3C)$	$\frac{1}{4}(3S+3P+D+3C)$	$\frac{1}{4}(3S-P+D-C)$
	**(vuw)**	$\frac{1}{2}(-S+D)$	$\frac{1}{2}(P-3C)$	$\frac{1}{2}(-3S-D)$	$\frac{1}{2}(P+C)$
	**(vwu)**	$\frac{1}{4}(S+P-D-3C)$	$\frac{1}{4}(-3S+P+3D-3C)$	$\frac{1}{4}(3S+3P+D+3C)$	$\frac{1}{4}(-3S+P-D+C)$
	**(wuv)**	$\frac{1}{4}(S-P-D+3C)$	$\frac{1}{4}(3S+P-3D-3C)$	$\frac{1}{4}(3S-3P+D-3C)$	$\frac{1}{4}(3S+P+D+C)$
	**(wvu)**	$\frac{1}{4}(S-P-D+3C)$	$\frac{1}{4}(-3S-P+3D+3C)$	$\frac{1}{4}(3S-3P+D-3C)$	$\frac{1}{4}(-3S-P-D-C)$
**(nml)**	**(uvw)**	$\frac{1}{2}(-S+D)$	$\frac{1}{2}(-P+3C)$	$\frac{1}{2}(3S+D)$	$\frac{1}{2}(P+C)$
	**(uwv)**	$\frac{1}{4}(S+P-D-3C)$	$\frac{1}{4}(3S-P-3D+3C)$	$\frac{1}{4}(-3S-3P-D-3C)$	$\frac{1}{4}(-3S+P-D+C)$
	**(vuw)**	$\frac{1}{2}(-S+D)$	$\frac{1}{2}(P-3C)$	$\frac{1}{2}(3S+D)$	$\frac{1}{2}(-P-C)$
	**(vwu)**	$\frac{1}{4}(S+P-D-3C)$	$\frac{1}{4}(-3S+P+3D-3C)$	$\frac{1}{4}(-3S-3P-D-3C)$	$\frac{1}{4}(3S-P+D-C)$
	**(wuv)**	$\frac{1}{4}(S-P-D+3C)$	$\frac{1}{4}(3S+P-3D-3C)$	$\frac{1}{4}(-3S+3P-D+3C)$	$\frac{1}{4}(-3S-P-D-C)$
	**(wvu)**	$\frac{1}{4}(S-P-D+3C)$	$\frac{1}{4}(-3S-P+3D+3C)$	$\frac{1}{4}(-3S+3P-D+3C)$	$\frac{1}{4}(3S+P+D+C)$

Table S1B. Transformation of the orientational order parameters S, P, D, C, under a permutation $\pi \in S_3$ of the principal axes, or permutation $\rho \in S_3$ of the reference axes. Permutations are shown in one-line notation (i.e., second row of Cauchy's two-line notation).

S1J Relation between biaxial order parameters and invariants of moment tensors.

We quantified orientational order of nematic tensors by the four classical order parameters S, P, D, C, as well as by co-orientational order parameters co-S, co-P, co-D, co-C. We present yet a third variant to quantify biaxial order: invariants of moment tensors [38].

We consider the first two moments, $\boldsymbol{T}$ and $\boldsymbol{V}$, of a distribution of nematic tensors $\boldsymbol{A}$

$$\begin{aligned} &\text{first moment:} && \boldsymbol{T} = \langle \boldsymbol{A} \rangle \quad , \\ &\text{second moment:} && \boldsymbol{V} = \langle \boldsymbol{A} \cdot \boldsymbol{A} \rangle \text{ with components } V_{\alpha\beta} = \langle A_{\alpha\gamma} A_{\gamma\beta} \rangle \quad . \end{aligned} \tag{S12}$$

Here, the dot operator '$\cdot$' corresponds to a matrix multiplication, i.e., $\boldsymbol{A} \cdot \boldsymbol{A}$ is again a 3×3-matrix with components $(\boldsymbol{A} \cdot \boldsymbol{A})_{\alpha\beta} = \sum_{\gamma=1}^{3} A_{\alpha\gamma} A_{\gamma\beta}$ for $\alpha, \beta = 1, \ldots, 3$. From these averaged tensors, we obtain scalar invariants by tensor contraction

$$\begin{aligned} I_1 &= \operatorname{tr} \boldsymbol{A}, & I_2 &= \operatorname{tr} \boldsymbol{A}^2, & I_3 &= \operatorname{tr} \boldsymbol{A}^3, \\ I_4 &= \operatorname{tr} \boldsymbol{V}, & I_5 &= \operatorname{tr} \boldsymbol{V}^2, & I_6 &= \operatorname{tr} \boldsymbol{V}^3, \quad \ldots \quad . \end{aligned} \tag{S13}$$

Note that since $\boldsymbol{A}$ is traceless, all non-zero contractions of the rank-4 super-tensor $\langle A_{\alpha\gamma} A_{\delta\beta} \rangle$ can already be derived from the rank-2 tensor $\boldsymbol{V}$.

If the ensemble of tensors $\boldsymbol{A}^{(i)}$ exhibits D_{2h}-symmetry, the moment tensors $\boldsymbol{T}$ and $\boldsymbol{V}$ diagonalize in a common eigenframe [39]. The invariants $I_1, \ldots, I_6$ can then be expressed in terms of symmetric polynomials in the eigenvalues of these tensors. Specifically, we denote the eigenvalues of $\boldsymbol{T}$ by μ_1, μ_2 and μ_3, and the eigenvalues of $\boldsymbol{V}$ by ν_1, ν_2, ν_3. Then the invariants of tensor moments are given as

$$\begin{aligned} I_1 &= \sum_{i=1}^{3} \mu_i = 0, & I_2 &= \sum_{i=1}^{3} \mu_i^2, & I_3 &= \sum_{i=1}^{3} \mu_i^3, \\ I_4 &= \sum_{i=1}^{3} \nu_i, & I_5 &= \sum_{i=1}^{3} \nu_i^2, & I_6 &= \sum_{i=1}^{3} \nu_i^3 \quad , \ \ldots \ . \end{aligned} \tag{S14}$$

Conversely, given the invariants I_1, I_2, ..., I_6, we can compute the eigenvalues μ_1, μ_2, μ_3, and ν_1, ν_2, ν_3, yet only up to a permutation, by solving the polynomial system of equations, Eq. (S14).

We now show how the tensor invariants are related to the classical order parameters S, P, D, C. We make the simplifying assumption that all nematic tensors $\boldsymbol{A}$ of the ensemble have identical eigenvalues α_1, α_2, α_3. As usual, we assume $\alpha_1 \geq \alpha_3 \geq \alpha_2$, and denote the corresponding eigenvectors as $\mathbf{n} = \mathbf{a}_2$, $\mathbf{m} = \mathbf{a}_1$, $\mathbf{l} = \mathbf{a}_3$. By Eq. (4), each tensor $\boldsymbol{A}$ is now associated with tensors $\boldsymbol{Q}$ and $\boldsymbol{B}$. We can write

$$\boldsymbol{A} = \xi_0\,\boldsymbol{Q} + \xi_1\,\boldsymbol{B} \quad , \tag{S15}$$

with weights ξ_0, ξ_1 that satisfy $\alpha_2 = \xi_0$, $\alpha_1 = -(\xi_0 + 3\xi_1)/2$, $\alpha_3 = -(\xi_0 - 3\xi_1)/2$. Note that a permutation of eigenvalues also changes the weights ξ_0 and ξ_1. Our usual ordering of eigenvalues with $\alpha_1 \geq \alpha_3 \geq \alpha_2$ corresponds to $0 \geq \xi_0/3 \geq \xi_1$. In the general case, where the eigenvalues of $\boldsymbol{A}$ vary within the ensemble, the invariants I_j will depend on both the orientational order of the principal axes of $\boldsymbol{A}$, as well as on the distribution of weights.

In the case of constant weights ξ_0, ξ_1, it follows $\boldsymbol{T} = \langle \boldsymbol{A} \rangle = \xi_0\,\langle \boldsymbol{Q} \rangle + \xi_1\,\langle \boldsymbol{B} \rangle$. Likewise, the second moment $\boldsymbol{V}$ can be expressed as a linear superposition of $\langle \boldsymbol{Q} \rangle$, $\langle \boldsymbol{B} \rangle$, and $\mathbb{1}$ as

$$\boldsymbol{V} = \zeta_0\,\langle \boldsymbol{Q} \rangle + \zeta_1\,\langle \boldsymbol{B} \rangle + \zeta_c\,\mathbb{1} \quad , \tag{S16}$$

where $\zeta_0 = (\xi_0^2 - 3\xi_1^2)/2$, $\zeta_1 = -\xi_0\xi_1$, $\zeta_c = (\xi_0^2 + 3\xi_1^2)/2$.

We thus have a direct correspondence between the eigenvalues of the moment tensors and the orientational order parameters S, P, D, C

$$\begin{aligned}
2\,\mu_1 &= \quad -\xi_0 S + \xi_0 P - \xi_1 D + 3\,\xi_1 C \;, \\
2\,\mu_2 &= \quad -\xi_0 S - \xi_0 P - \xi_1 D - 3\,\xi_1 C \;, \\
2\,\mu_3 &= \quad 2\xi_0 S + 2\xi_1 D \;, \\
2\,\nu_1 &= 2\zeta_c - \zeta_0 S + \zeta_0 P - \zeta_1 D + 3\,\zeta_1 C \;, \\
2\,\nu_2 &= 2\zeta_c - \zeta_0 S - \zeta_0 P - \zeta_1 D - 3\,\zeta_1 C \;, \\
2\,\nu_3 &= 2\zeta_c + 2\zeta_0 S + 2\zeta_1 D \;.
\end{aligned} \tag{S17}$$

Note $\mu_1 + \mu_2 + \mu_3 = 0$, while $\nu_1 + \nu_2 + \nu_3 = 3\zeta_c$.

Together, Eq. (S14) and Eq. (S17) allow to compute the orientational order parameters S, P, D, C from the invariants of tensor moments. Note that the first

tensor moment is not sufficient to determine the OOP, but that at least the second moment is needed. (In the non-generic case $\xi_0 = -1$ and $\xi_1 = \pm 1$ for which $\zeta_0 = \xi_0$ and $\zeta_1 = \xi_1$, also a third tensor moment needs to be taken into account.)

We emphasize that the values of the invariants I_1, I_2, ..., I_6 are independent of any ordering of axes, whereas the values of the orientational order parameters S, P, D, C depend on the ordering of both the principal and the reference axes. This is reflected in Eq. (S14) by the fact that $I_1, I_2, \ldots, I_6$ do not change under neither a permutation of the eigenvalues μ_1, μ_2, μ_3, nor a permutation of the eigenvalues ν_1, ν_2, ν_3. In contrast, Eq. (S17) shows that S, P, D, C depend on the ordering of eigenvalues. As a consequence, the orientational order parameters S, P, D, C can change discontinuously if system parameters are smoothly varied, while the invariants I_1, I_2, ..., I_6 do not, see Fig S1H. Despite this desirable property of the invariants I_1, I_2, ..., I_6, the invariants lack the intuitive geometric interpretation of the orientational order parameters S, P, D, C. The co-orientational order parameters co-S, co-P, co-D, co-C introduced in the main text combine the advantageous property of a smooth dependence on system parameters with intuitive geometric interpretation.

S1K Commented computer program. A commented Matlab script, which computes COOP for prototypical examples, is available online (written in Matlab, The Mathworks Inc.; compatible with GNU Octave). The program generates synthetic data sets of tripods of nematic axes using inverse sampling of a Boltzmann distribution for different effective interaction energies H, as used in Fig 3, Fig 5A, Fig S1F and Fig S1H, panel B. Three-dimensional visualization can be rotated interactively by the user.

Additional references cited in S1 Appendix

38. Scholich A. Biaxial Nematic Order in Liver Tissue. PhD thesis. 2018, TU Dresden.

39. Matteis GD. Continuum Landau model for biaxial nematic liquid crystals. Mol Cryst Liq Cryst. 2009;500(1):31–50.

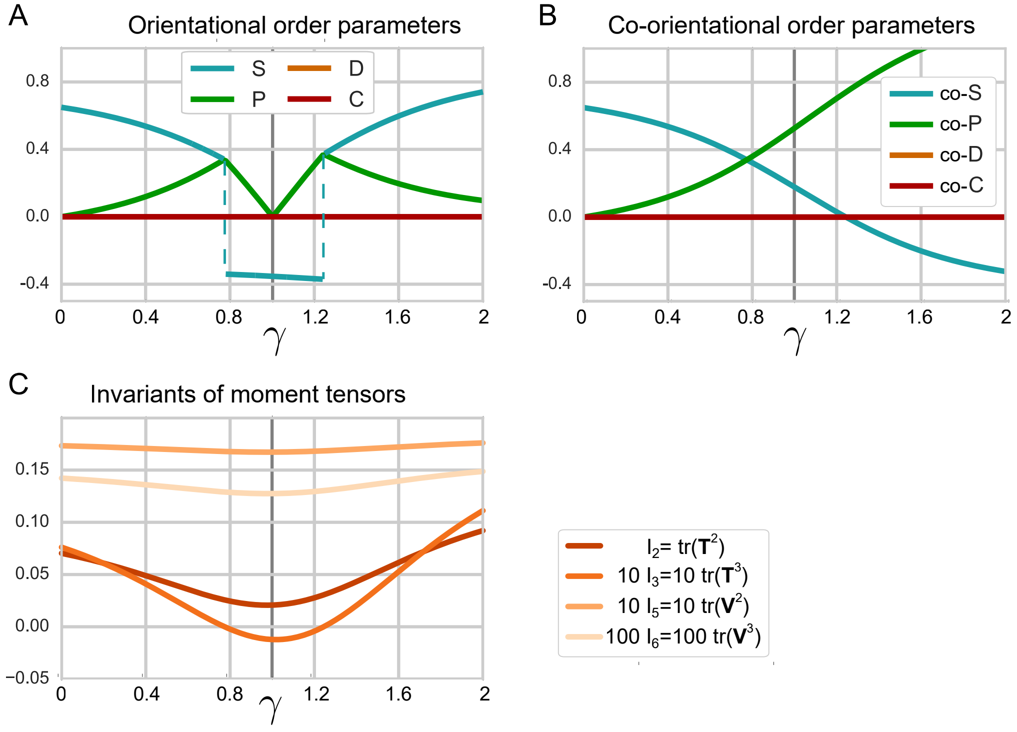

Fig S1H. Comparison of orientational order parameters (OOP), co-orientational order parameters (COOP), and invariants of moment tensors for phase-biaxial order. (A) Orientational order parameters S, P, D, C for a Boltzmann distribution $p \sim \exp(-H)$ of biaxial objects governed by the dimensionless Hamiltonian $H = -5(\mathbf{a}_2 \cdot \mathbf{e}_2)^2 - 5\gamma\,(\mathbf{a}_2 \cdot \mathbf{e}_1)^2$ as function of the effective interaction parameter γ. The ordering π of principal axes with $\mathbf{n} = \mathbf{a}_{\pi(2)}$ as well as the ordering ρ of reference axes with $\mathbf{w} = \mathbf{e}_{\rho(2)}$, $\mathbf{v} = \mathbf{e}_{\rho(3)}$, $\mathbf{u} = \mathbf{e}_{\rho(1)}$ in the definition of S, P, D, C, Eq. (7) is chosen such that $|S|$ is maximal and $P \geq 0$, $C \geq 0$ (as common in the field of liquid crystals [23]). Note the discontinuous change of S and P caused by a change in ρ. (B) Same as panel A for the co-orientational order parameters co-S, co-P, co-D, co-C, where a fixed ordering $\pi = \mathrm{id}$ of principal axes and a fixed ordering $\rho = \mathrm{id}$ of reference axes is used. For this choice, Eq. (9) holds for $\gamma = 0$, but not for general γ. (C) Invariants of tensor moments as defined in Eq. (S14) for the same system.
